# Transcription factors involved in retinogenesis are co-opted by the circadian clock following photoreceptor differentiation

**DOI:** 10.1242/dev.104380

**Published:** 2014-07

**Authors:** Ricardo Laranjeiro, David Whitmore

**Affiliations:** Centre for Cell and Molecular Dynamics, Department of Cell and Developmental Biology, University College London, London WC1E 6DE, UK

**Keywords:** Circadian clock, *neurod*, Photoreceptors, Retina, Transcription factors, Zebrafish

## Abstract

The circadian clock is known to regulate a wide range of physiological and cellular processes, yet remarkably little is known about its role during embryo development. Zebrafish offer a unique opportunity to explore this issue, not only because a great deal is known about key developmental events in this species, but also because the clock starts on the very first day of development. In this study, we identified numerous rhythmic genes in zebrafish larvae, including the key transcriptional regulators *neurod* and *cdx1b*, which are involved in neuronal and intestinal differentiation, respectively. Rhythmic expression of *neurod* and several additional transcription factors was only observed in the developing retina. Surprisingly, these rhythms in expression commenced at a stage of development after these transcription factors are known to have played their essential role in photoreceptor differentiation. Furthermore, this circadian regulation was maintained in adult retina. Thus, once mature photoreceptors are formed, multiple retinal transcription factors fall under circadian clock control, at which point they appear to play a new and important role in regulating rhythmic elements in the phototransduction pathway.

## INTRODUCTION

The circadian clock is a highly conserved timekeeping system that allows organisms to anticipate and take advantage of daily environmental changes. Circadian clocks are self-sustained molecular oscillators, based on transcription-translation negative-feedback loops, that are entrained to local time by the environmental light-dark (LD) cycle. Zebrafish have become an important model system in which to study vertebrate circadian biology owing to the highly decentralised nature of its circadian system. Zebrafish tissues and cell lines contain endogenous circadian oscillators that are themselves light responsive, allowing for the direct entrainment of the cellular clock by external LD cycles, even *in vitro* (Whitmore et al., 2000; Vallone et al., 2004; Carr and Whitmore, 2005; Tamai et al., 2007). In the zebrafish embryo, robust oscillations in the expression of circadian clock genes can be seen on the second day of development, although the circadian pacemaker appears to begin even earlier, on the first day of development (Dekens and Whitmore, 2008). In fact, embryos are light responsive during the first stages of gastrulation, before the differentiation of classical light-responsive structures and only hours following fertilisation (Tamai et al., 2004).

Zebrafish are also a major model system for the study of vertebrate development. This is largely due to development occurring *ex vivo*, embryo transparency and amenability for large-scale mutant screens (Driever et al., 1996; Haffter et al., 1996). Consequently, a large number of genes crucial for the regulation of important developmental processes have been identified. In particular, this includes determinants of cell fate and transcription factors involved in cell differentiation within specific tissues, as well as signalling pathways involved in general organogenesis.

One aspect of biology that remains largely unexplored, however, is whether the circadian clock that is present within the developing embryo plays any role in the regulation and timing of the expression of these essential developmental factors. Does the circadian clock influence development, such that crucial developmental genes are expressed in a circadian fashion? If so, at what point during development does clock regulation begin? Does this regulation occur only in specific cells and tissues?

In this study, we identified numerous rhythmically expressed genes, which are involved in various developmental processes, such as the cell cycle, DNA repair, retinal/neural development and intestinal function. One of the most strongly rhythmic genes that we identified was *neurod*, and, somewhat to our surprise, its expression was only rhythmic within the developing retina. Moreover, several additional transcription factors involved in photoreceptor differentiation, including *crx*, *rx2* and *nr2e3*, also showed robust oscillations in the larval retina. The onset of this clock control occurs at a specific developmental stage and continues into adulthood, after photoreceptor differentiation in the retina has largely been completed and when the known function of these genes would have been completed. Numerous components of the phototransduction cascade in photoreceptors were also found to oscillate, clearly suggesting a role for the circadian clock in the regulation of photoreceptor function. Our hypothesis is that the circadian clock gains control of developmentally essential retinal transcription factors, after they have completed their role in cell differentiation, and then employs them in the control of stable retinal physiology.

## RESULTS

### Rhythmic gene expression during zebrafish larval development

Considering the ubiquitousness of circadian clocks, surprisingly little is known about their role during vertebrate development. This is in part due to the difficulty of studying early developmental events in mammals, whereas this is relatively straightforward in zebrafish. To address this issue, we performed a gene expression analysis during zebrafish development using the NanoString nCounter Analysis System, a digital quantification method whereby hundreds of target mRNAs can be analysed in a single reaction (Geiss et al., 2008). Zebrafish larvae were collected at four time points per day between 4 and 7 days post fertilisation (dpf). This developmental time window was chosen as most tissues and organs are present and functional but, at the same time, are still actively developing and maturing. Moreover, at these stages, the circadian clock is fully functional, and clock-controlled processes, such as cell cycle timing (Dekens et al., 2003; Laranjeiro et al., 2013) and locomotor activity rhythms (Hurd and Cahill, 2002), become evident. Zebrafish larvae raised on an LD cycle were compared with those raised in constant light (LL), a condition known to repress clock function in zebrafish (Tamai et al., 2007). Larvae raised on an LD cycle until 6 dpf and then transferred to constant darkness (DD) on day 7 were also analysed to assess whether genes that oscillated maintained their rhythmicity under free-running, constant conditions. For this quantitative expression analysis, 87 genes were selected, comprising circadian clock, cell cycle and DNA repair components and cell lineage/tissue-specific genes, including those known to be expressed in brain/neuron/glia, eye, intestine, pancreas, kidney, liver, heart, skeletal muscle, blood cells, vasculature and bone (the complete list is included in supplementary material Fig. S1).

**Fig. 1.**
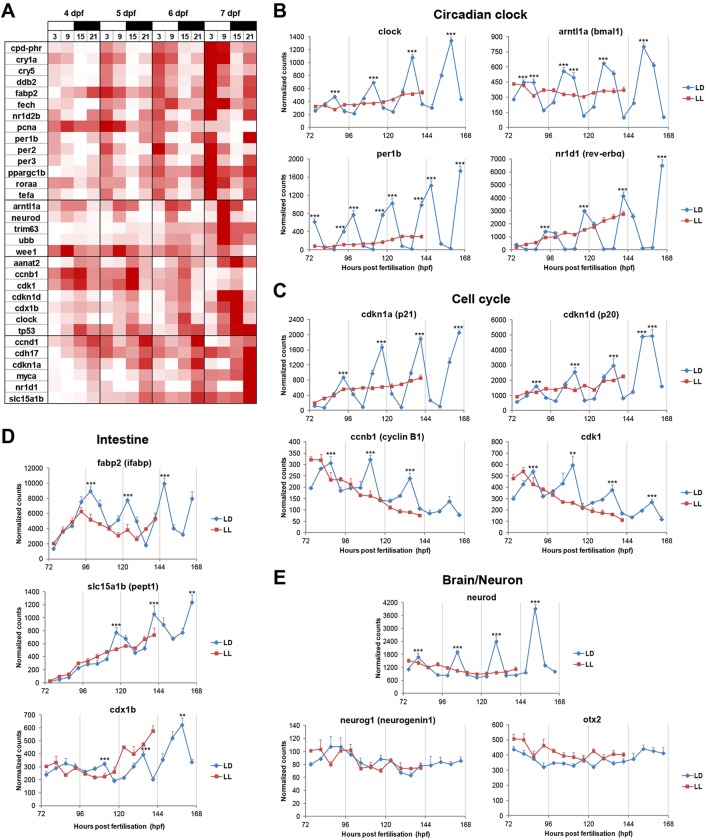
**Rhythmic gene expression during zebrafish larval development.** (A) NanoString results summarised in a heat map, which includes all the genes that exhibited rhythmic expression on an LD cycle between 4 and 7 dpf (*n*=3). Expression values are presented in a colour range from white (lowest) to red (highest). Genes were grouped in four categories based on the time of day of their expression peak: ZT3, ZT9, ZT15 or ZT21 (from top to bottom, respectively). White and black bars above the heat map represent light and dark phases, respectively. (B-E) NanoString analysis of circadian clock (B), cell cycle (C), intestine (D) and brain/neuron (E) genes during zebrafish development (*n*=3). Gene expression on an LD cycle (4-7 dpf) was compared with that in LL conditions (4-6 dpf). Statistically significant differences between the expression peak and trough on each day (Fisher's LSD test) are indicated: **P<0.01, ***P<0.001. Error bars indicate s.e.m.

As expected, core circadian clock genes [including *clock*, *arntl1a* (*bmal1*), *per1b*, *per3* and *nr1d1* (*rev-erbα*)] exhibited a clear 24 h rhythm on an LD cycle and an arrhythmic or ‘flat’ expression profile in LL. Importantly, these rhythms were maintained after transfer into DD, confirming regulation by the circadian clock as opposed to being directly light driven (Fig. 1A,B; supplementary material Fig. S1). One important output of the circadian clock is the regulation of cell cycle timing, and in this zebrafish developmental analysis we identified several rhythmically expressed genes involved in regulating different phases of the cell cycle: *ccnd1*, *cdkn1a* (*p21*), *cdkn1d* (*p20*) (G1/S transition); *myca* (*c-myc*), *pcna* (S phase); and *ccnb1*, *cdk1*, *wee1* (G2/M transition) (Fig. 1A,C; supplementary material Fig. S1). This suggests that circadian clock regulation of the cell cycle can occur at multiple levels, resulting in complex interactions between these two cellular oscillators.

The three DNA repair genes analysed [*cry5* (*6-4 photolyase*), *cpd-phr* (*zgc:66475*) and *ddb2*] exhibited robust rhythms on an LD cycle, with the peak of expression occurring during the light phase. However, when transferred into DD, these rhythms were completely abolished or significantly damped, showing that light rather than the circadian clock is the primary driver of these rhythms. Consistent with this observation, the expression levels of these genes in LL were constitutively high (supplementary material Fig. S1). Other previously identified light-regulated genes had a similar expression profile, including *per2*, *cry1a*, *tefa* and *fech* (supplementary material Fig. S1) (Gavriouchkina et al., 2010; Weger et al., 2011).

Interestingly, three intestinal-specific genes included in this analysis seemed to be clock regulated, as shown by their rhythmicity on an LD cycle and in DD (Fig. 1A,D; supplementary material Fig. S1): intestinal fatty acid binding protein (*fabp2*, or *ifabp*) is involved in fat absorption and metabolism; solute carrier family 15 member 1b (*slc15a1b*, or *pept1*) plays an important role in the uptake of oligopeptides by intestinal cells; and caudal type homeobox 1b (*cdx1b*), which is a ParaHox transcription factor, is essential for the regulation of proliferation and differentiation of various zebrafish intestinal cell lineages (Flores et al., 2008; Chen et al., 2009). These results not only suggest an active role for the circadian clock in the regulation of intestinal function, but also provide a potential molecular link (i.e. *cdx1b*) between the clock, the cell cycle and intestinal differentiation.

The most striking 24 h expression rhythm from all the cell lineage/tissue-specific genes was observed for *neurod*. This proneural basic helix-loop-helix (bHLH) transcription factor exhibited sharp upregulation in expression in the late light phase, at zeitgeber time (ZT) 9, where ZT0 refers to lights on. Moreover, *neurod* expression is completely arrhythmic in LL, and the rhythmicity observed under LD conditions is maintained in DD, showing clear circadian clock regulation. Other brain/neuron-specific transcription factors (e.g. *neurog1* and *otx2*) did not display any circadian regulation (Fig. 1A,E; supplementary material Fig. S1).

The results of this gene expression analysis during zebrafish larval development show that the circadian clock has multiple targets in cell cycle regulation, may affect the function of specific tissues (e.g. intestine), and controls the expression of essential transcription factors (e.g. *cdx1b* and *neurod*) involved in the differentiation of specific cell lineages.

### *neurod* rhythmic expression is restricted to retinal photoreceptors

**Fig. 2.**
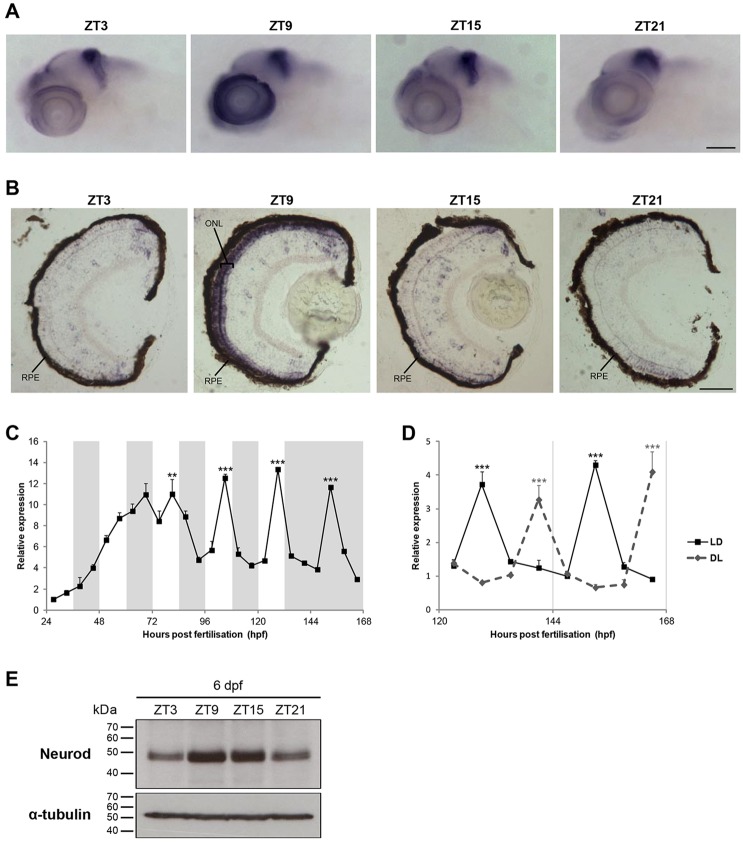
***neurod* rhythmic expression is restricted to retinal photoreceptors.** (A,B) Representative images at four time points of *neurod* WISH on 6 dpf larvae (A) and of *in situ* hybridisation for *neurod* on 6 dpf larval eye sections (B). ZT, zeitgeber time; RPE, retinal pigment epithelium; ONL, outer nuclear layer. (C) qPCR analysis of *neurod* expression in zebrafish larvae raised on an LD cycle until 6 dpf and then transferred to DD on day 7 (*n*=3). White and grey backgrounds represent light and dark phases, respectively. (D) qPCR analysis of *neurod* expression in 6-7 dpf larvae raised on an LD cycle or a DL cycle (*n*=3). Statistically significant differences between the expression peak and trough on each day (Fisher's LSD test) are indicated: **P<0.01, ***P<0.001. Error bars indicate s.e.m. (E) Representative western blots of Neurod and α-tubulin expression on 6 dpf larvae at four time points. Scale bars: 100 µm in A; 50 µm in B.

Neurod is a bHLH transcription factor that plays a role in cell cycle exit, cell fate determination, differentiation and cell survival. In vertebrates, *neurod* is expressed in areas of the brain, such as the cortex, cerebellum, eye, olfactory bulb and midbrain, but also in non-neuronal tissues, such as the endocrine pancreas (Chae et al., 2004). The very dramatic and precisely timed daily rhythm in *neurod* expression observed in our developmental analysis, and the crucial role it plays in determining neuronal cell fate, led us to focus on *neurod*. The initial aim was to determine where this transcription factor is rhythmically expressed during larval development, so we performed whole-mount *in situ* hybridisation (WISH) on 6 dpf larvae. *neurod* expression was exclusively detected in the head region, specifically in the brain and eye. Whereas the mid-hindbrain boundary exhibited constitutively high levels throughout the LD cycle, the larval eye specifically showed a clear upregulation of *neurod* expression at ZT9 (Fig. 2A), consistent with the expression peak observed in the NanoString analysis.

Previous reports have shown that, in the zebrafish retina, *neurod* is expressed in the rod and cone photoreceptor lineages (Ochocinska and Hitchcock, 2007; Nelson et al., 2008) and is essential to promote photoreceptor progenitor withdrawal from the cell cycle, leading to terminal differentiation (Ochocinska and Hitchcock, 2009). Therefore, we hypothesised that rhythmic *neurod* expression in the larval eye could be responsible for the rhythmic differentiation of photoreceptor cells. To address this, 5 dpf larvae were exposed to a BrdU pulse and then allowed to develop for a further 48 h. Two days after the BrdU pulse, almost every BrdU-positive cell was still in the stem cell region of the retina, termed the circumferential marginal zone (CMZ), and, consequently, BrdU-positive photoreceptor cells were rarely, if ever, detected (supplementary material Fig. S2A,B). Given that photoreceptor differentiation is extremely limited at these stages of development, it is highly unlikely that this process is the main target of *neurod* rhythmicity. In fact, and rather surprisingly, *in situ* hybridisation on larval sections revealed that rhythmic expression of *neurod* is not found in retinal progenitors, but rather in the retinal outer nuclear layer (ONL), which corresponds to the photoreceptor cell layer (Fig. 2B). These results indicate that the circadian rhythm in *neurod* expression is only seen in differentiated photoreceptors. We therefore examined, using qPCR, when *neurod* rhythmicity becomes established during zebrafish development. As shown in Fig. 2C, *neurod* expression levels steadily increased from 24 to 72 h post fertilisation (hpf). On the fourth day of development, the first peak in expression at ZT9 was observed, and in the following days the amplitude of this rhythm became stronger. We conclude that the *neurod* expression rhythm only appears in zebrafish development after the wave of differentiation of rod and cone photoreceptors, which occurs between 48 and 72 hpf (Branchek and Bremiller, 1984; Raymond et al., 1995; Schmitt and Dowling, 1996).

We also compared *neurod* expression between larvae raised on an LD cycle with those raised on a reverse, dark-light (DL) cycle. If *neurod* is truly clock regulated, we would expect these rhythms to be 12 h out of phase (anti-phase) between the two lighting conditions. As shown in Fig. 2D, this was exactly what we observed. Importantly, a western blot analysis on 6 dpf larvae revealed that *neurod* expression is not only rhythmic at the transcript level but also at the protein level, with the peak of Neurod occurring at ZT9-15 (Fig. 2E).

Together, these results reveal that, following the wave of photoreceptor differentiation between 2 and 3 dpf, *neurod* becomes strongly rhythmic, indicating that the circadian clock is the primary regulator of *neurod* transcription at this time, promoting a 24 h expression rhythm that is restricted to differentiated photoreceptors.

### Additional retinal transcription factors are rhythmically expressed in differentiated photoreceptors

The transcription factors that are expressed by rod and cone lineages during photoreceptor differentiation are well-characterised in the zebrafish retina. We asked whether clock regulation was *neurod* specific or if it could be extended to additional photoreceptor transcriptional regulators. Cone-rod homeobox (*crx*), retinal homeobox 1 (*rx1*) and retinal homeobox 2 (*rx2*) are expressed in both rod and cone lineages at different stages of photoreceptor genesis (Chuang et al., 1999; Shen and Raymond, 2004; Bernardos et al., 2007; Nelson et al., 2008, 2009). Neural retina leucine zipper (*nrl*) and photoreceptor-specific nuclear receptor (*nr2e3*) are only expressed in rods of the mature retina, but during development in zebrafish, unlike in other vertebrates, they can be expressed in both rod and cone progenitors (Chen et al., 2005; Nelson et al., 2008).

**Fig. 3.**
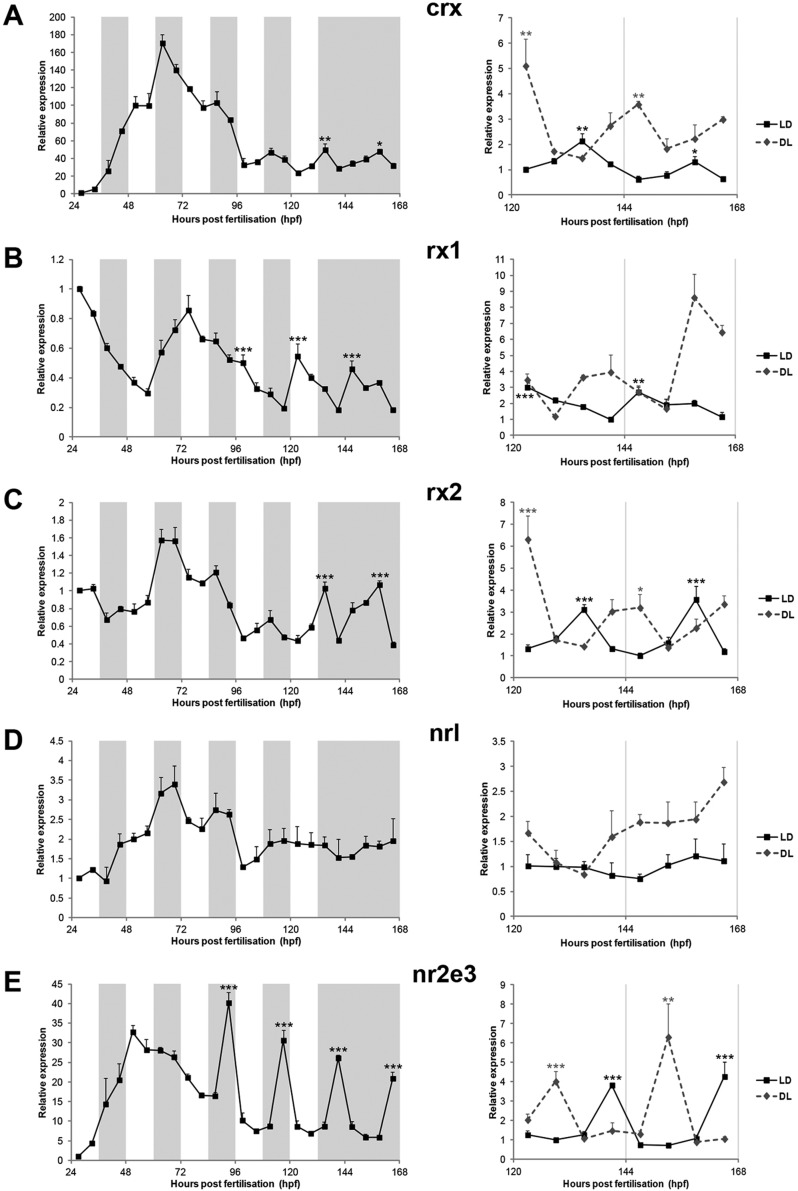
**Additional retinal transcription factors are rhythmically expressed during zebrafish development.** (A-E) qPCR analysis of *crx* (A), *rx1* (B), *rx2* (C), *nrl* (D) and *nr2e3* (E) expression during zebrafish development. Expression analysis was performed on larvae raised on an LD cycle until 6 dpf and then transferred to DD on day 7 (left) or on 6-7 dpf larvae raised either on an LD or a DL cycle (right) (*n*=3-5). White and grey backgrounds represent light and dark phases, respectively. Statistically significant differences between the expression peak and trough on each day (Fisher's LSD test) are indicated: *P<0.05, **P<0.01, ***P<0.001. Error bars indicate s.e.m.

Using qPCR, we analysed the expression of these five transcription factors during zebrafish development. On the second and third day of development (24-72 hpf), none of these genes was rhythmic and, in general, their expression levels increased with time (Fig. 3A-E), consistent with the role played by these transcription factors during the wave of photoreceptor differentiation (48-72 hpf). After 72 hpf, however, a significant decrease in expression was observed, followed by the emergence of a circadian rhythm for all genes, except *nrl*, the expression of which was fairly constant from 5 dpf onwards. The 24 h expression rhythms appeared on the fourth or fifth day of development and persisted in DD (at 7 dpf), although the peak of expression occurred at different times of the day for different genes (Fig. 3A-E). Circadian clock involvement was further tested by comparing larvae raised on an LD cycle versus a DL cycle. *crx*, *rx2* and *nr2e3* showed a clear anti-phase expression rhythm when exposed to a DL cycle, confirming clock regulation. By contrast, *rx1* expression on a DL cycle was not clearly rhythmic, suggesting that not only the circadian clock but also developmental pathways might be controlling its expression (Fig. 3A-E).

**Fig. 4.**
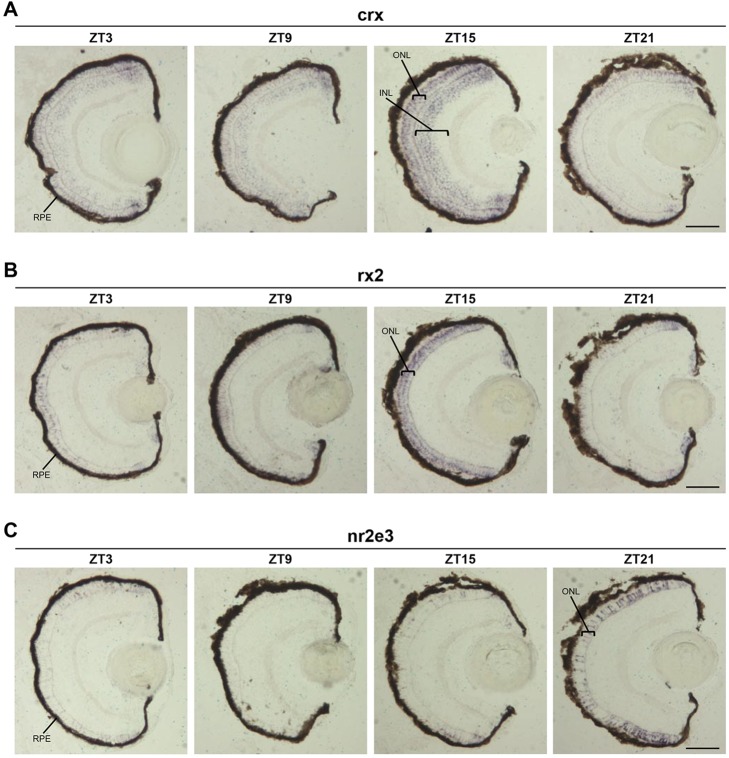
**Circadian expression of retinal transcription factors in differentiated photoreceptors.** (A-C) Representative images of *in situ* hybridisation for *crx* (A), *rx2* (B) and *nr2e3* (C) on 6 dpf larval eye sections at four time points. RPE, retinal pigment epithelium; INL, inner nuclear layer; ONL, outer nuclear layer. Scale bars: 50 µm.

Expression analysis by WISH on 6 dpf larvae revealed that *crx*, *rx1*, *rx2* and *nr2e3* are predominantly expressed in the larval eye, with some expression in the developing brain. Conversely, *nrl* expression was stronger throughout the brain than in the eye (supplementary material Fig. S3A-E). To determine exactly where the retinal transcription factors that exhibited a clear circadian regulation (i.e. *crx*, *rx2* and *nr2e3*) are expressed in the eye, we performed *in situ* hybridisation on larval sections. Rhythmic expression of *crx* was found in the ONL (photoreceptor layer) and the outer part of the inner nuclear layer (INL) of the larval eye (Fig. 4A). *rx2* rhythmic expression was restricted to the ONL (Fig. 4B). *nr2e3* rhythmic expression was also observed in the ONL; however, not every photoreceptor cell expressed this transcription factor. In the central retina, *nr2e3* expression was restricted to scattered photoreceptors, a spatial and temporal pattern previously described for rod photoreceptors (Fadool, 2003). In the less mature retinal periphery, *nr2e3*-expressing cells were present at a higher density and are therefore likely to include some cone precursors (Chen et al., 2005) (Fig. 4C).

These results reveal for the first time that several retinal transcription factors involved in photoreceptor generation during the first days of zebrafish development subsequently become strongly rhythmically expressed in differentiated photoreceptors. In addition, they suggest a more global regulation by the circadian clock of photoreceptor gene expression and, possibly, of photoreceptor function.

### Circadian expression of retinal transcription factors is maintained in adult zebrafish

**Fig. 5.**
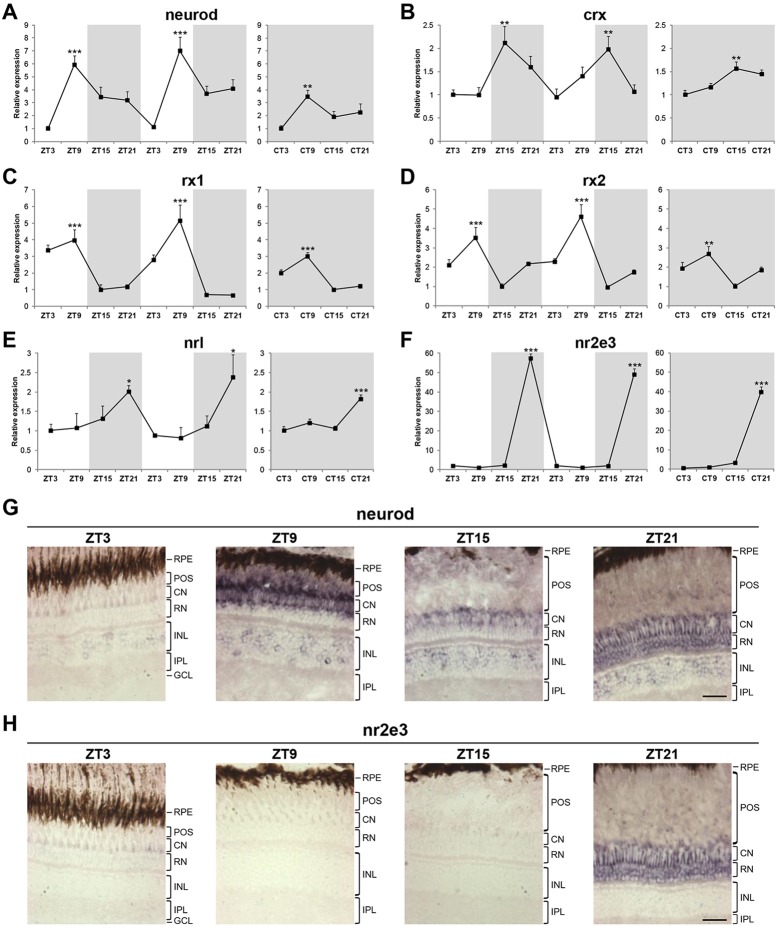
**Circadian expression of retinal transcription factors is maintained in adult zebrafish.** (A-F) qPCR analysis of *neurod* (A), *crx* (B), *rx1* (C), *rx2* (D), *nrl* (E) and *nr2e3* (F) expression in adult eyes over two days of an LD cycle and one subsequent day of DD (*n*=3-4). Zeitgeber time (ZT) or circadian time (CT) indicates the four time points analysed per day. White and grey backgrounds represent light and dark phases, respectively. Statistically significant differences between the expression peak and trough on each day (Fisher's LSD test) are indicated: *P<0.05, **P<0.01, ***P<0.001. Error bars indicate s.e.m. (G,H) Representative images of *in situ* hybridisation for *neurod* (G) and *nr2e3* (H) on adult eye sections at four time points. Note that expression in the photoreceptors at ZT21 is restricted to rods for both *neurod* and *nr2e3*. The signal present in the layer of cone nuclei corresponds to rod inner segments that connect the rod nuclei to the rod outer segments. RPE, retinal pigment epithelium; POS, photoreceptor outer segments; CN, cone nuclei; RN, rod nuclei; INL, inner nuclear layer; IPL, inner plexiform layer; GCL, ganglion cell layer. Scale bars: 30 µm.

To determine if circadian expression of retinal transcription factors is maintained in adult zebrafish, we performed a qPCR analysis on adult eyes collected over two days of an LD cycle and one subsequent day of DD. All transcription factors exhibited rhythmic expression on an LD cycle, and the rhythm was maintained in DD, although with a lower amplitude for most genes (Fig. 5A-F). The amplitude of rhythmic expression on an LD cycle varied between two- and sevenfold for all transcription factors, except *nr2e3*, which showed a remarkably high amplitude of at least 50-fold.

*In situ* hybridisation performed on adult eye sections allowed us to identify the retinal cell types that rhythmically expressed the six transcription factors in question owing to the characteristic layered organisation of the zebrafish adult retina (Branchek and Bremiller, 1984) and by comparison with *in situ* signals of known rod and cone markers, specifically the α-subunit of rod and cone transducins (supplementary material Fig. S4A,B). *neurod* expression was predominantly found in the photoreceptor layer, although some positive cells were also observed in the INL at all time points analysed. The peak in expression observed at ZT9 was restricted to cone photoreceptors. Interestingly, at the end of the dark phase (at ZT21), *neurod* expression was only observed in rod photoreceptors, showing that *neurod* is rhythmically expressed by both rod and cone photoreceptors but at different times of the day (Fig. 5G). *crx* peak expression occurred at ZT15 in two different regions: the outer part of the INL and, more noticeably, in cone photoreceptors (supplementary material Fig. S5A). *rx1* also showed rhythmic expression in different cell types at different times of the day; at ZT3, *rx1* was predominantly expressed in the INL and rod nuclei, whereas at ZT9, expression was restricted to cone photoreceptors (supplementary material Fig. S5B). Rhythmic expression of *rx2* was observed in the INL and rod nuclei, with the peak at ZT9 (supplementary material Fig. S5C). Expression of *nrl* and *nr2e3* was restricted to rod photoreceptors and exhibited an expression peak at ZT21 (Fig. 5H; supplementary material Fig. S5D).

**Fig. 6.**
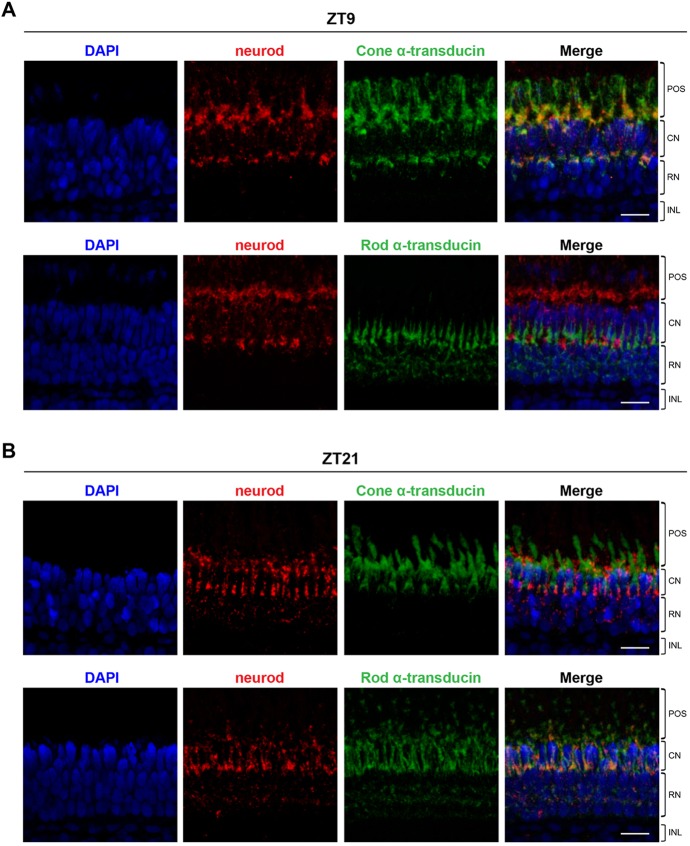
**Dynamic expression of *neurod* in zebrafish adult photoreceptors.** Representative maximum projection confocal images of double fluorescent *in situ* hybridisation for *neurod* and cone α-transducin (*gnat2*) or *neurod* and rod α-transducin (*gnat1*) on adult eye sections at ZT9 (A) and ZT21 (B). DAPI was used as a nuclear counterstain. POS, photoreceptor outer segments; CN, cone nuclei; RN, rod nuclei; INL, inner nuclear layer. Scale bars: 15 µm.

To confirm the identity of the photoreceptor cells expressing some of the rhythmic transcription factors, double fluorescent *in situ* hybridisations were performed on adult eye sections. As shown in Fig. 6A, *neurod* expression was restricted to cone photoreceptors at ZT9 as assessed by colocalisation with cone α-transducin fluorescent signals and by the absence of colocalisation of *neurod* and rod α-transducin fluorescent signals. Conversely, at ZT21, *neurod* expression only colocalised with rod α-transducin expression (Fig. 6B), confirming that *neurod* is dynamically regulated in the zebrafish adult retina. Expression of *crx* at ZT15 was confirmed to occur in cone photoreceptors by predominant colocalisation with cone α-transducin fluorescent signals (supplementary material Fig. S6).

These results reveal that circadian expression of retinal transcription factors is established during larval development and maintained in adult zebrafish. However, this process seems to be refined in the adult retina, resulting in an even more complex expression pattern that includes rhythmic expression of the same transcription factor in different cell types at different times of the day (as summarised in Table 1).

**Table 1.**
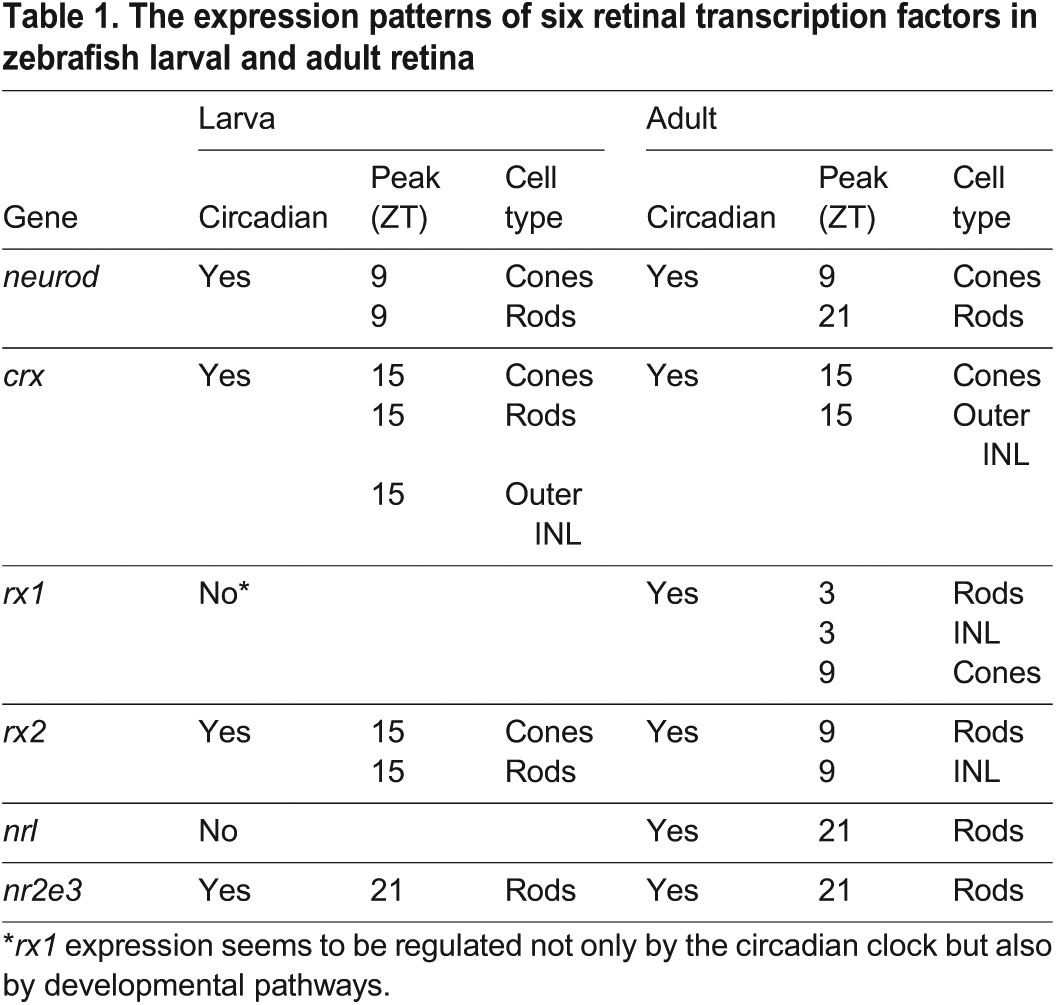
**The expression patterns of six retinal transcription factors in zebrafish larval and adult retina**

### Components of the photoreceptor phototransduction cascade are also rhythmically expressed in the zebrafish retina

The clock-controlled transcription factors identified in this study have the potential to control numerous target genes, implying a wider circadian control of photoreceptor gene expression as well as photoreceptor function. The main role of retinal photoreceptors is the conversion of a light signal into an electrical signal, a process called phototransduction. The phototransduction cascade involves multiple proteins, including opsins, transducins, phosphodiesterases (PDEs), cyclic nucleotide-gated (CNG) ion channels, G protein-coupled receptor kinases (GRKs) and arrestins (Fu and Yau, 2007; Larhammar et al., 2009).

**Fig. 7.**
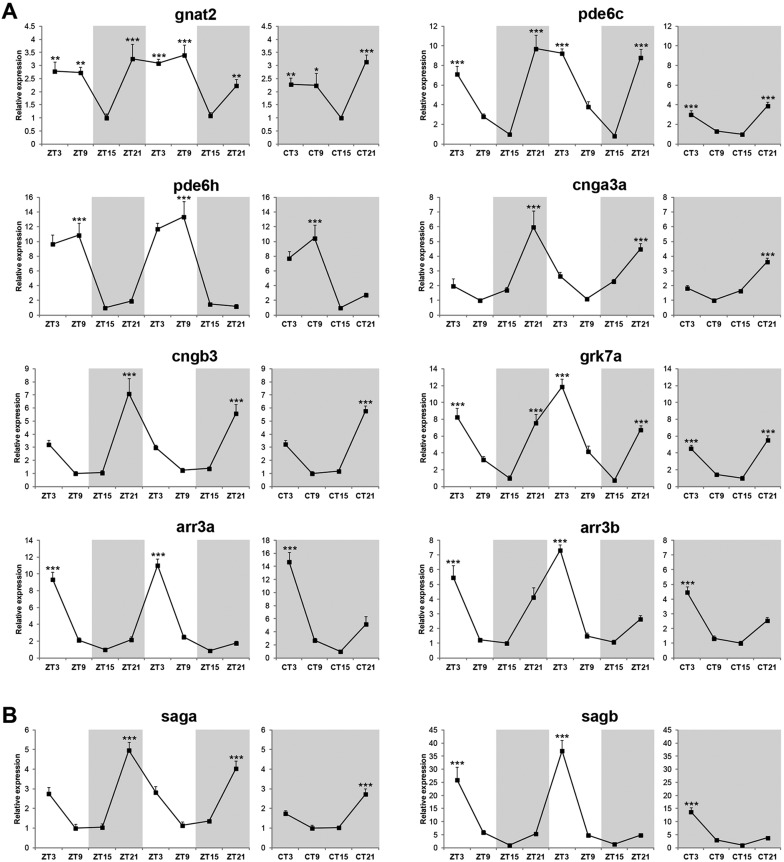
**Phototransduction genes are also rhythmically expressed in the zebrafish adult retina.** qPCR analysis of cone-specific (A) and rod-specific (B) phototransduction genes in zebrafish adult eyes over two days of an LD cycle and one subsequent day of DD (*n*=3-4). Zeitgeber time (ZT) or circadian time (CT) indicates the four time points analysed per day. White and grey backgrounds represent light and dark phases, respectively. Statistically significant differences between the expression peak and trough on each day (Fisher's LSD test) are indicated: *P<0.05, **P<0.01, ***P<0.001. Error bars indicate s.e.m.

**Fig. 8.**
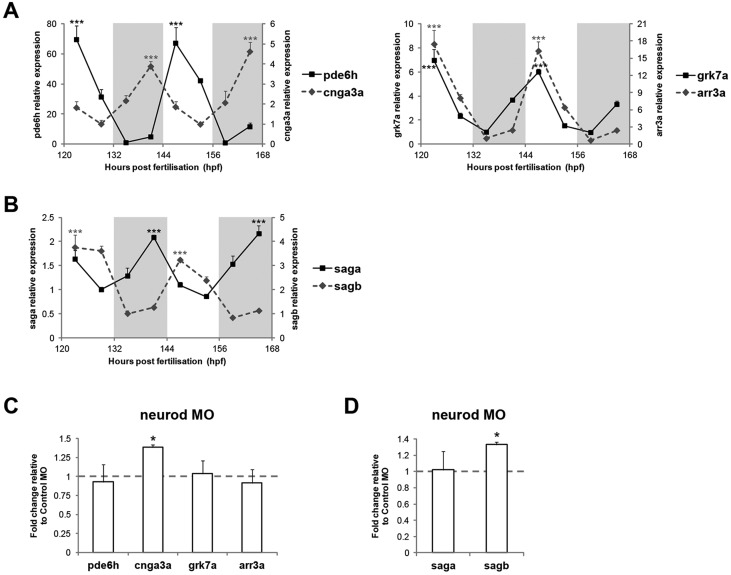
**Regulation of phototransduction gene expression during zebrafish development.** (A,B) qPCR analysis of cone-specific (A) and rod-specific (B) phototransduction genes on 6-7 dpf larvae raised on an LD cycle (*n*=3). White and grey backgrounds represent light and dark phases, respectively. Statistically significant differences between the expression peak and trough on each day (Fisher's LSD test) are indicated: ***P<0.001. (C,D) qPCR analysis of cone-specific (C) and rod-specific (D) phototransduction genes on 6 dpf larval eyes injected with *neurod* vivo-morpholino or control vivo-morpholino (*n*=3). Larval eyes were collected at ZT3 (C) or ZT15 (D). *P<0.05 (Student's *t*-test). Error bars indicate s.e.m.

We performed a qPCR analysis on zebrafish adult eyes to assess whether components of the phototransduction cascade are rhythmically expressed. Cone- and rod-specific genes were selected for this analysis (Table 2). Several cone-specific phototransduction genes exhibited a clear circadian rhythm in LD and DD conditions, including transducin (*gnat2*, *gnb3a*), PDE (*pde6c*, *pde6h*), CNG ion channel (*cnga3a*, *cnga3b*, *cngb3*), GRK (*grk7a*) and arrestin (*arr3a*, *arr3b*) genes (Fig. 7A; supplementary material Fig. S7A,B). By contrast, from all the rod-specific components analysed, only arrestin genes (*saga*, *sagb*) showed an obvious rhythmic expression profile in both LD and DD conditions (Fig. 7B; supplementary material Fig. S8). These results show that key components of the phototransduction cascade in cone and rod photoreceptors are rhythmically expressed in the zebrafish adult retina, which makes them likely target genes of the transcription factors identified in this study. Consistent with this idea was the observation that both cone- and rod-specific phototransduction genes were also rhythmically expressed in 6-7 dpf larvae (Fig. 8A,B), a developmental time when most retinal transcription factors already show circadian regulation.

**Table 2.**
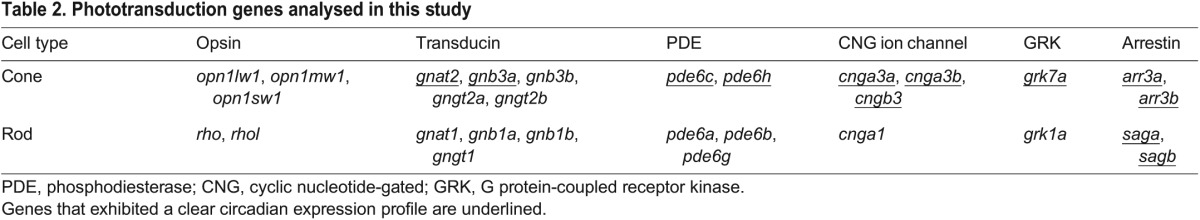
**Phototransduction genes analysed in this study**

To further explore the hypothesis that the rhythmic retinal transcription factors control the expression of phototransduction genes, we used a translation-blocking vivo-morpholino to knockdown Neurod expression in the larval retina and then assess if phototransduction gene expression was affected. Larval eyes injected with *neurod* vivo-morpholino exhibited consistent upregulation of *cnga3a*, a cone-specific CNG ion channel, and of *sagb*, a rod-specific arrestin, whereas the expression of other phototransduction components was unaffected relative to control morpholino injections (Fig. 8C,D). Moreover, *cnga3a* and *sagb* promoters (3 kb) contain four and one Neurod binding sites, respectively, as predicted by MatInspector (core similarity >0.70, optimised matrix similarity). These results suggest that Neurod contributes to regulating the expression of some phototransduction genes, both in rod and cone photoreceptors.

Together, our results support the hypothesis that genes crucial for neural/retinal development become strongly clock controlled at later developmental stages and in the adult retina, where they play a key role in the circadian clock regulation of photoreceptor function, in particular in the detection and transmission of light signals.

## DISCUSSION

Circadian clock function in vertebrates has been primarily studied in adult organisms and in specific adult tissues. By contrast, our understanding of the influence of circadian clocks on vertebrate development is very limited. In this study, we performed a gene expression analysis during zebrafish larval development using NanoString technology, which led to the identification of numerous rhythmically expressed genes involved in various biological processes, including cell cycle regulation, DNA damage repair, tissue-specific functions and cell differentiation. Furthermore, we described novel circadian clock regulation of multiple retinal transcription factors, which becomes established during zebrafish larval development and is maintained in the adult retina. This regulation was primarily observed in rod and cone photoreceptors, suggesting an active role for these transcription factors in the clock-controlled expression of phototransduction components, leading to the rhythmic regulation of photoreceptor function.

Cell division occurs preferentially at specific times of the day in most organisms, in a process known to be regulated by the circadian clock (Johnson, 2010; Masri et al., 2013). Here, we identified rhythmically expressed cell cycle regulators involved in the various phases of the cell cycle. These results confirmed and expanded our previous studies of zebrafish clock-controlled cell cycle timing (Tamai et al., 2012; Laranjeiro et al., 2013). The CDK inhibitors *cdkn1a* and *cdkn1d* are directly clock controlled (Laranjeiro et al., 2013), whereas other cell cycle components might be indirectly clock controlled or simply rhythmically expressed as a consequence of a cell cycle rhythm promoted by other genes.

Circadian expression of cell lineage/tissue-specific genes was also found in our NanoString analysis. In particular, clock-controlled expression of intestinal absorption genes (*fabp2* and *slc15a1b*) suggest that intestinal function in zebrafish larval development is already circadian regulated, a phenomenon known to occur in the adult mammalian intestine (Pácha and Sumova, 2013). Furthermore, the transcription factor Cdx1b, which is the functional equivalent of mammalian Cdx2 and essential to regulate the proliferation and differentiation of several intestinal cell lineages, is likely to provide a direct link during zebrafish larval development between the circadian clock, the cell cycle and cell differentiation. Given the perpetual self-renewal/regenerative nature of the intestinal epithelium, it will be interesting to determine whether clock regulation of *cdx1b* is maintained in the adult intestine.

*neurod* was one of the most strongly rhythmic genes identified in the NanoString analysis and became the focus of our study. We described, for the first time, that *neurod* mRNA and protein are very strongly rhythmically expressed in the zebrafish retina. This observation might explain some of the discrepancies in *neurod* expression reported previously (Ochocinska and Hitchcock, 2007; Nelson et al., 2008; Thomas et al., 2012), since its expression is highly dependent on the time of the day at which samples are collected, and whether the circadian clock is entrained or not. The ‘adult-like’ pattern of *neurod* expression at 96 hpf previously described by Ochocinska and Hitchcock (2007) is identical to the expression pattern that we observed for 6 dpf larvae at ZT3, ZT15 and ZT21. However, its expression pattern is completely different at ZT9, when all differentiated rods and cones express *neurod*, demonstrating the importance of taking circadian time into account when performing developmental studies. Furthermore, both rod and cone photoreceptors express *neurod* in the adult retina, but at different times of the day. This highly dynamic and complex regulation of *neurod* transcripts might also explain the differences in the expression levels of endogenous *neurod* and reporter EGFP observed by Thomas et al. (2012) in the adult zebrafish retina.

We have identified several additional retinal transcription factors that are clock controlled. Rhythmic expression of *crx*, *rx2* and *nr2e3* became apparent during zebrafish development, but only after the wave of photoreceptor differentiation (48-72 hpf). *nrl* transcription is clock controlled in the adult retina, but no rhythmicity was found during development. This might suggest that some retinal genes only become regulated by the circadian clock in the fully mature zebrafish retina. The absence of *nrl* rhythmic expression in zebrafish larvae might also be explained by the fact that whole larvae RNA was used for qPCR analysis. As determined by WISH, *nrl* expression is most abundant in the developing brain; therefore, it is possible that larval eyes exhibit a rhythmic *nrl* expression, which is masked by abundant, non-rhythmic expression in the brain. Previous studies have suggested that expression of both *rx1* and *rx2* in the zebrafish adult retina is restricted to the CMZ and cone photoreceptors (Chuang et al., 1999; Raymond et al., 2006). Here, we show that *rx1* expression can, in fact, be found in the INL and rods at ZT3 and in cones at ZT9, and that *rx2* expression can be observed in the INL and rods at ZT9. These discrepancies can be explained, at least in part, by the previously unknown rhythmic expression of these genes and the different times of the day at which expression analyses were performed.

The nuclear receptor Nr2e3 exhibited the most impressive clock-controlled transcriptional rhythm in zebrafish adult retina, with an amplitude of at least 50-fold on an LD cycle. High amplitudes such as this are rarely seen outside the core circadian genes. Since nuclear receptors, such as Nr1d1, have already been shown to have an important role in the circadian clock mechanism, this raises the intriguing possibility that Nr2e3 might also be involved in the circadian transcription-translation feedback loops. Previous reports in mouse retina have identified an interaction between Nr1d1 and Nr2e3, and several genes involved in photoreceptor development and function that are co-targeted by these nuclear receptors (Cheng et al., 2004; Mollema et al., 2011). It will be interesting to determine if a similar phenomenon occurs in zebrafish rod photoreceptors and whether some of the target genes are components of the core clock mechanism.

All the retinal transcription factors identified in this study as being clock controlled have a role in photoreceptor differentiation in the first days of zebrafish development. Subsequent to photoreceptor differentiation, these transcription factors then become rhythmically expressed, suggesting an alternative role for these genes in photoreceptor maturation, maintenance and/or function. In fact, even though the wave of photoreceptor generation is largely completed by 72 hpf, the postmitotic rods and cones undergo full maturation in the following days and weeks, including the onset of expression of photoreceptor-specific genes, growth of photoreceptor outer segments (POSs) and expansion of synaptic connectivity (Branchek and Bremiller, 1984; Biehlmaier et al., 2003; Morris and Fadool, 2005). The highly dynamic transcriptional regulation observed in the zebrafish adult retina is likely to contribute to the control of day and night vision, as mediated by cone and rod photoreceptors, respectively.

Genome-wide expression studies have identified thousands of genes (including a high percentage of transcription factors) that show circadian oscillations in at least one tissue; however, the number of genes showing circadian regulation in multiple tissues is extremely low (Panda et al., 2002; Miller et al., 2007; Yan et al., 2008). These results strongly suggest a hierarchical organisation in which core clock components directly regulate the expression of a few tissue-specific transcription factors, which can then control numerous target genes by a transcriptional cascade. This type of circadian gene regulatory network has recently been proposed to exist in zebrafish (Li et al., 2013). Therefore, it is likely that a similar phenomenon is also present in the zebrafish retina, where key components of the phototransduction pathway might be regulated by clock-controlled, tissue-specific transcription factors. The six clock-controlled retinal transcription factors identified in this study (*neurod*, *crx*, *rx1*, *rx2*, *nrl* and *nr2e3*) are obvious candidates to regulate phototransduction gene expression. In fact, downregulation of Neurod in the larval eye led to a significant increase in *cnga3a* and *sagb* expression, pointing to a repressor activity of Neurod on some phototransduction genes. Neurod might act directly as a transcriptional repressor (Itkin-Ansari et al., 2005) or might indirectly control phototransduction gene expression by inducing an intermediate transcriptional repressor. Although our results suggest that Neurod alone can only regulate a limited number of phototransduction components, it is likely that the other clock-controlled transcription factors and different combinations thereof could account for most, if not all, rhythmic regulation of phototransduction gene expression. Significantly, in mammals, Crx, Nrl and Nr2e3 have been shown to regulate the transcription of several phototransduction genes, including *Gnat2*, *Gnb3*, *Pde6c*, *Pde6h*, *Cngb3* and *Arr3* (Sakuma et al., 1998; Zhu et al., 2002; Chen et al., 2005; Peng et al., 2005; Peng and Chen, 2005; Corbo et al., 2010; Hao et al., 2012), all of which exhibit circadian expression in zebrafish retina.

The vertebrate retina is a remarkably rhythmic tissue, with many cellular, biochemical and physiological processes following a circadian rhythm, including visual sensitivity and electroretinogram responses, disc shedding of POSs, melatonin synthesis and dopamine release (Iuvone et al., 2005). Nonetheless, the molecular regulation underlying these circadian processes is largely unknown. Our findings shed light on the possible transcriptional cascades involved in these clock-controlled retinal functions and reveal that key developmental transcription factors are co-opted into this process.

## MATERIALS AND METHODS

### Zebrafish lines and maintenance

The zebrafish line AB/TL was used for all experiments. Adult zebrafish were maintained under standard conditions in the UCL fish facility at 28.5°C on an LD cycle (14 h light:10 h dark). Zebrafish embryos were obtained from natural spawning, transferred to 25 cm^2^ tissue culture flasks in egg water, and incubated in a large-volume water bath at 28°C on an LD cycle (12 h light:12 h dark), LL or DD for up to 7 dpf. All experiments were conducted in accordance with the United Kingdom Animals (Scientific Procedures) Act 1986.

### NanoString nCounter analysis system

Zebrafish larvae were harvested from 4 to 7 dpf in TRIzol (three independent biological replicates) and total RNA was isolated following the manufacturer's instructions (Ambion). Gene-specific probes were designed and synthesized by NanoString Technologies. The nCounter gene expression assay was performed by UCL Genomics, Scientific Support Services. Data were normalised using the internal positive controls included by NanoString Technologies, and the geometric mean of *actin, beta 1* (*actb1*, NM_131031.1) and *eukaryotic translation* *elongation factor 1α, like 1* (*eef1a1l1*, NM_131263.1) was used to control for total RNA input across samples. CodeSet information and complete gene expression data are available from the Dryad Digital Repository: http://doi.org/10.5061/dryad.r07bc.

### Quantitative PCR (qPCR)

Zebrafish embryos/larvae, larval or adult eyes were harvested in TRIzol. RNA extraction, reverse transcription and qPCR were carried out as described (Tamai et al., 2012). *eef1a1l1* was used as a reference gene. Primer sequences are listed in supplementary material Table S1.

### Colorimetric *in situ* hybridisations

The following digoxigenin (DIG)-labelled riboprobes were used for colorimetric *in situ* hybridisations: *neurod* (Blader et al., 1997), *crx* (Liu et al., 2001), *rx1*, *rx2* (Chuang et al., 1999), *nrl* (Nelson et al., 2008), *nr2e3* (Chen et al., 2005), rod α-transducin (*gnat1*) and cone α-transducin (*gnat2*).

WISH on 6 dpf larvae was performed according to standard protocols (Thisse and Thisse, 2008). BM purple (Roche) was used as substrate.

Adult eyes or 6 dpf larvae were harvested and 10 µm cryosections prepared as described (Laranjeiro et al., 2013). *In situ* hybridisation on sections was performed as previously described (Schaeren-Wiemers and Gerfin-Moser, 1993) with the following modifications: blocking was performed with 10% goat serum in buffer B1; incubation with anti-DIG-AP antibody (Roche, 11093274910; 1:5000) was performed overnight at 4°C; to perform the colour reaction, BCIP/NBT (Vector Laboratories) in buffer B3/0.1% Tween 20 was used. All sections were stained with DAPI to aid identification of the retinal cell layers.

### Fluorescent *in situ* hybridisations

Double fluorescent *in situ* hybridisations were performed with a combination of DIG-labelled (*neurod* and *crx*) and fluorescein-labelled (*gnat1* and *gnat2*) riboprobes. The protocol was similar to that described in the previous section for cryosections, with the following modifications: incubation at 4°C overnight with anti-fluorescein-POD antibody (Roche, 11426346910; 1:2000) was followed by incubation for 1 h at room temperature in TSA Plus Fluorescein Solution (PerkinElmer); inactivation of the first peroxidase was for 30 min in methanol/3% H_2_O_2_; incubation at 4°C overnight with anti-DIG-POD antibody (Roche, 11093274910; 1:2000) was followed by incubation for 1 h at room temperature in TSA Plus Cyanine 5 Solution (PerkinElmer). Sections were imaged on a Leica TCS SPE confocal microscope.

### BrdU labelling and immunohistochemistry

At 5 dpf, larvae were incubated at ZT3 for 40 min in BrdU solution (10 mM BrdU and 5% DMSO in egg water) at 28°C. After BrdU exposure, larvae were transferred to 10 mM thymidine in egg water to develop for a further 48 h. At 7 dpf, larvae were harvested and cryosections prepared as described (Laranjeiro et al., 2013). Sections were first processed for photoreceptor staining with mouse zpr-1 or zpr-3 (Zebrafish International Resource Center; 1:100) and goat anti-mouse conjugated to Alexa 594 (Molecular Probes, A11005; 1:100) antibodies, fixed in 4% paraformaldehyde for 1 h, and then processed for BrdU staining as described (Laranjeiro et al., 2013). Sections were imaged on a Leica DMLB fluorescence microscope.

### Western blot

6 dpf larvae were harvested in Laemmli buffer. Western blots were performed as described (Laranjeiro et al., 2013) using mouse anti-Neurod1 (Abcam, ab60704; 1:1000) (Kroehne et al., 2011), horse anti-mouse-HRP (Cell Signaling, 7076; 1:1000), rat anti-α-tubulin (AbD Serotec, MCA77G; 1:1500), and goat anti-rat-HRP (Calbiochem, 401416; 1:1000).

### Morpholino injections

Intraocular injections were performed in anaesthetised 5 dpf larvae with 0.4 nl/eye 0.5 mM *neurod* translation-blocking vivo-morpholino (5′-TGAC-TTCGTCATGTCGGAACTCTAG-3′) (Sarrazin et al., 2006; Ochocinska and Hitchcock, 2009) or control vivo-morpholino (5′-CCTCTTACC-TCAGTTACAATTTATA-3′) (Gene Tools). Injections were performed 24-27 h before dissection of larval eyes.

### Statistical analysis

The data in this study are presented as mean±s.e.m. (*n*≥3). Genes were considered to be rhythmically expressed if the same rhythmic expression profile was observed on at least 2 consecutive days (i.e. expression peak/trough occurring at the same time of the day), and if statistically significant differences were found on each day. Statistical significance was determined by one-way ANOVA on each day (*P*<0.05), followed by Fisher's least significant difference (LSD) test. For simplicity, only significant differences between the expression peak and the expression trough on each day are shown in the figures.

For morpholino experiments, statistical significance was determined by two-tailed Student's *t*-test.

## Supplementary Material

Supplementary Material
