# Supplementary material for: Transcription factors involved in retinogenesis are co-opted by the circadian clock following photoreceptor differentiation
Source: Development. 2014 Jul;141(13):2644–56. doi: 10.1242/dev.104380 (PMC4146392; doi:10.1242/dev.104380)
Supplement: Supplementary Material [file supp_141_13_2644__index.html]

Transcription factors involved in retinogenesis are co-opted by the circadian clock following photoreceptor differentiation — Supplementary Material 

# Transcription factors involved in retinogenesis are co-opted by the circadian clock following photoreceptor differentiation

## DEV104380 Supplementary Material

**Files in this Data Supplement:**

- **Supplementary Material**
