# Supplementary material for: Transcription factors involved in retinogenesis are co-opted by the circadian clock following photoreceptor differentiation
Source: Development. 2014 Jul;141(13):2644–56. doi: 10.1242/dev.104380 (PMC4146392; doi:10.1242/dev.104380)
Supplement: Supplementary Material [file supp_141.13.2644_DEV104380.pdf]

**Fig. S1. NanoString analysis of gene expression during zebrafish larval development.** Heat map of all genes included in the NanoString analysis (n=3, ordered alphabetically). For each gene, three different lighting conditions are presented: light-dark cycle from 4 to 7 dpf (LD); constant light from 4 to 6 dpf (LL); light-dark cycle until 6 dpf and then transferred to constant darkness on day 7 (DD). Expression values are presented in a colour range from white (lowest) to red (highest). Grey bars represent time points not included in our analysis.

**Fig. S2. Limited photoreceptor differentiation after 5 days of zebrafish development.** (**A,B**) Representative cross-sections of 7 dpf larval eyes (48 hours after BrdU pulse) immunostained for BrdU and the cone marker zpr-1 (**A**) or BrdU and the rod marker zpr-3 (**B**). DAPI was used as a nuclear counterstain. CMZ, circumferential marginal zone. Scale bars: 50 µm.

**Fig. S3. Expression of retinal transcription factors in zebrafish larvae.** (**A-E**) Representative images of WISH for *crx* (**A**), *rx1* (**B**), *rx2* (**C**), *nrl* (**D**), and *nr2e3* (**E**) on 6 dpf larvae at four different time points. Scale bars: 100 µm.

**Fig. S4. Expression of rod and cone photoreceptor markers in adult retina. (A,B)** Representative images of *in situ* hybridisation for rod α-transducin (*gnat1*) (**A**) and cone α-transducin (*gnat2*) (**B**) on adult eye sections at four different time points. The expression pattern of each marker varies considerably throughout the day due to the positional changes of photoreceptor outer segments (retinomotor movements). Note that the *gnat1* signal present in the layer of cone nuclei (CN) corresponds to rod inner segments that connect the rod nuclei (RN) to the rod outer segments. RPE, retinal pigment epithelium; POS, photoreceptor outer segments; CN, cone nuclei; RN, rod nuclei; INL, inner nuclear layer; IPL, inner plexiform layer; GCL, ganglion cell layer. Scale bars: 30 µm.

**Fig. S5. Expression of retinal transcription factors in zebrafish adult retina.** (**A-D**) Representative images of *in situ* hybridisation for *crx* (**A**), *rx1* (**B**), *rx2* (**C**), and *nrl* (**D**) on adult eye sections at four different time points. Note that expression of *nrl* at ZT21 is restricted to rod photoreceptors. The signal present in the layer of cone nuclei (CN) corresponds to rod inner segments that connect the rod nuclei (RN) to the rod outer segments. RPE, retinal pigment epithelium; POS, photoreceptor outer segments; CN, cone nuclei; RN, rod nuclei; INL, inner nuclear layer; IPL, inner plexiform layer; GCL, ganglion cell layer. Scale bars: 30 µm.

**Fig. S6. Expression of *crx* in zebrafish adult photoreceptors.** Representative maximum projection confocal images of double fluorescent *in situ* hybridisation for *crx* and cone α-transducin (*gnat2*) or *crx* and rod α-transducin (*gnat1*) on adult eye sections at ZT15. DAPI was used as a nuclear counterstain. POS, photoreceptor outer segments; CN, cone nuclei; RN, rod nuclei; INL, inner nuclear layer. Scale bars: 15 µm.

**Fig. S7. Expression of cone-specific phototransduction genes in zebrafish adult eye.** (**A**) qPCR analysis of cone-specific phototransduction genes in adult eyes over two days of a LD cycle and one subsequent day of DD (n=3-4). These two genes exhibited a clear circadian expression profile. (**B**) qPCR analysis of other cone-specific phototransduction genes that did not exhibit a clear circadian regulation in adult eyes (n=3-4). Note that some genes (e.g. *opn1lw1*, *gnb3b*, and *gngt2b*) appear to be rhythmically expressed on a LD cycle, but this rhythmicity is not maintained in DD. Zeitgeber time (ZT) or circadian time (CT) indicates the four time points analysed per day. White and grey backgrounds represent light and dark phases, respectively. Statistically significant differences between the expression peak and trough on each day (Fisher's LSD test) are indicated: *$P$<0.05, **$P$<0.01, ***$P$<0.001. Error bars indicate s.e.m.

**Fig. S8. Expression of rod-specific phototransduction genes in zebrafish adult eye.** qPCR analysis of rod-specific phototransduction genes that did not exhibit a clear circadian regulation in adult eyes (n=3-4). Note that some genes (e.g. *gnb1b*, and *gngt1*) appear to be rhythmically expressed on a LD cycle, but this rhythmicity is not maintained in DD. Zeitgeber time (ZT) or circadian time (CT) indicates the four time points analysed per day. White and grey backgrounds represent light and dark phases, respectively. Statistically significant differences between the expression peak and trough on each day (Fisher's LSD test) are indicated: *$P$<0.05, **$P$<0.01, ***$P$<0.001. Error bars indicate s.e.m.

**Fig. S1**

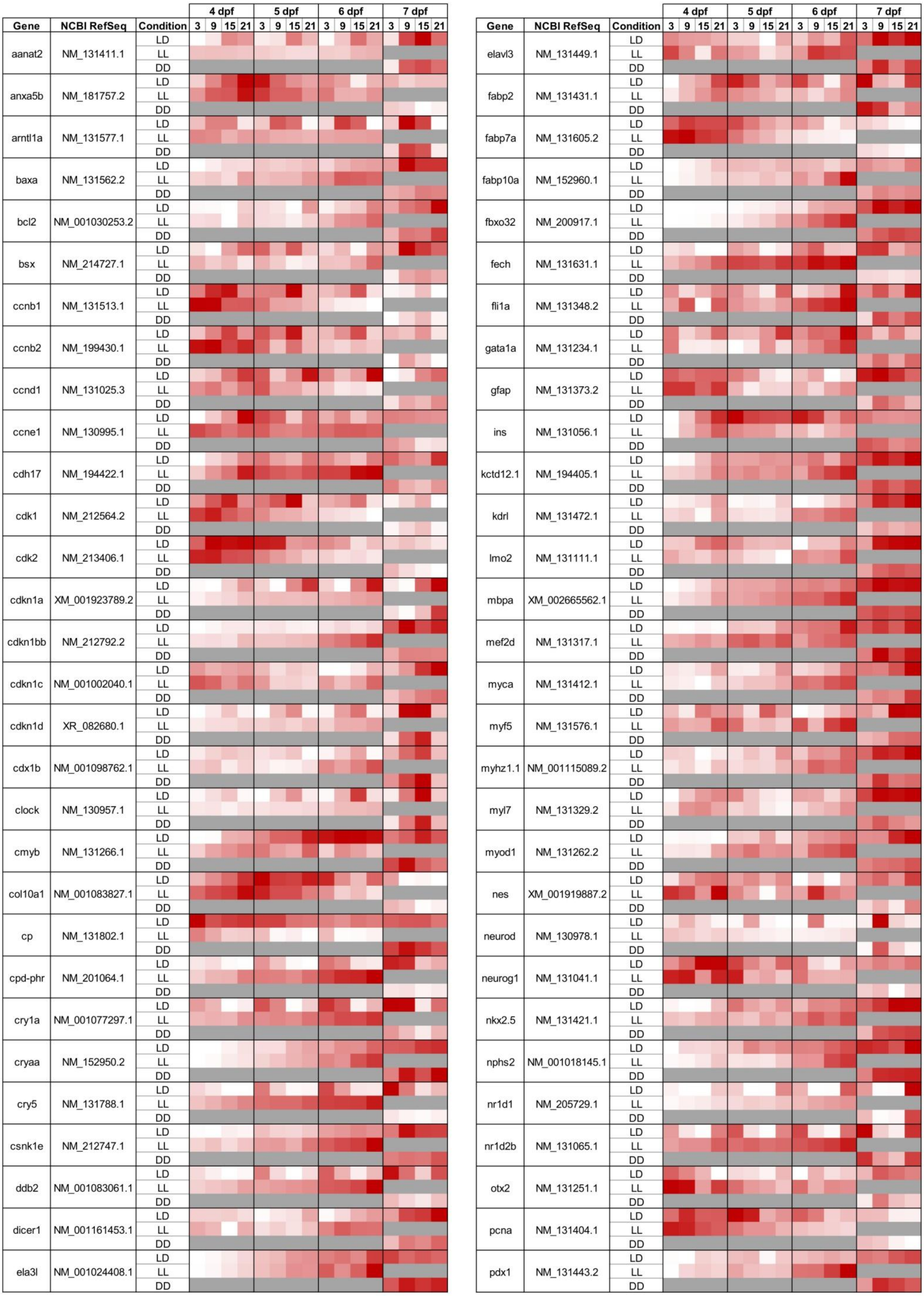
Gene
NCBI RefSeq
Condition
4 dpf
5 dpf
6 dpf
7 dpf
3 9 15 21
LD
LL
DD
aanat2 NM_131411.1
anxa5b NM_181757.2
arntl1a NM_131577.1
baxa NM_131562.2
bcl2 NM_001030253.2
bsx NM_214727.1
ccnb1 NM_131513.1
ccnb2 NM_199430.1
ccnd1 NM_131025.3
ccne1 NM_130995.1
cdh17 NM_194422.1
cdk1 NM_212564.2
cdk2 NM_213406.1
cdkn1a XM_001923789.2
cdkn1bb NM_212792.2
cdkn1c NM_001002040.1
cdkn1d XR_082680.1
cdx1b NM_001098762.1
clock NM_130957.1
cmyb NM_131266.1
col10a1 NM_001083827.1
cp NM_131802.1
cpd-phr NM_201064.1
cry1a NM_001077297.1
cryaa NM_152950.2
cry5 NM_131788.1
csnk1e NM_212747.1
ddb2 NM_001083061.1
dicer1 NM_001161453.1
ela3l NM_001024408.1
elavl3 NM_131449.1
fabp2 NM_131431.1
fabp7a NM_131605.2
fabp10a NM_152960.1
fbxo32 NM_200917.1
fech NM_131631.1
fli1a NM_131348.2
gata1a NM_131234.1
gfap NM_131373.2
ins NM_131056.1
kctd12.1 NM_194405.1
kdrl NM_131472.1
lmo2 NM_131111.1
mbpa XM_002665562.1
mef2d NM_131317.1
myca NM_131412.1
myf5 NM_131576.1
myhz1.1 NM_001115089.2
myl7 NM_131329.2
myod1 NM_131262.2
nes XM_001919887.2
neurod NM_130978.1
neurog1 NM_131041.1
nkx2.5 NM_131421.1
nphs2 NM_001018145.1
nr1d1 NM_205729.1
nr1d2b NM_131065.1
otx2 NM_131251.1
pcna NM_131404.1
pdx1 NM_131443.2

**Fig. S1 (Cont.)**

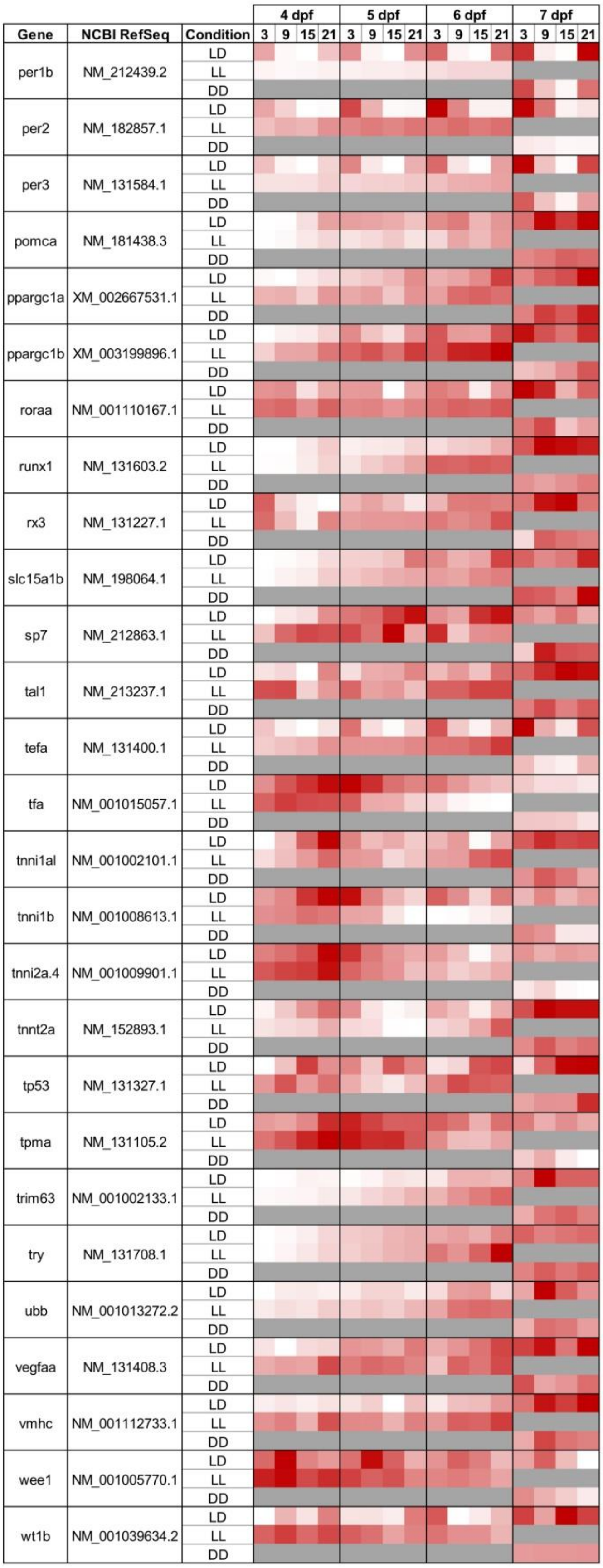
4 dpf
5 dpf
6 dpf
7 dpf
Gene
NCBI RefSeq
Condition
3 9 15 21
LD
LL
DD
per1b NM_212439.2
per2 NM_182857.1
per3 NM_131584.1
pomca NM_181438.3
ppargc1a XM_002667531.1
ppargc1b XM_003199896.1
roraa NM_001110167.1
runx1 NM_131603.2
rx3 NM_131227.1
slc15a1b NM_198064.1
sp7 NM_212863.1
tal1 NM_213237.1
tefa NM_131400.1
tfa NM_001015057.1
tnni1al NM_001002101.1
tnni1b NM_001008613.1
tnni2a.4 NM_001009901.1
tnnt2a NM_152893.1
tp53 NM_131327.1
tpma NM_131105.2
trim63 NM_001002133.1
try NM_131708.1
ubb NM_001013272.2
vegfaa NM_131408.3
vmhc NM_001112733.1
wee1 NM_001005770.1
wt1b NM_001039634.2

**Fig. S2**

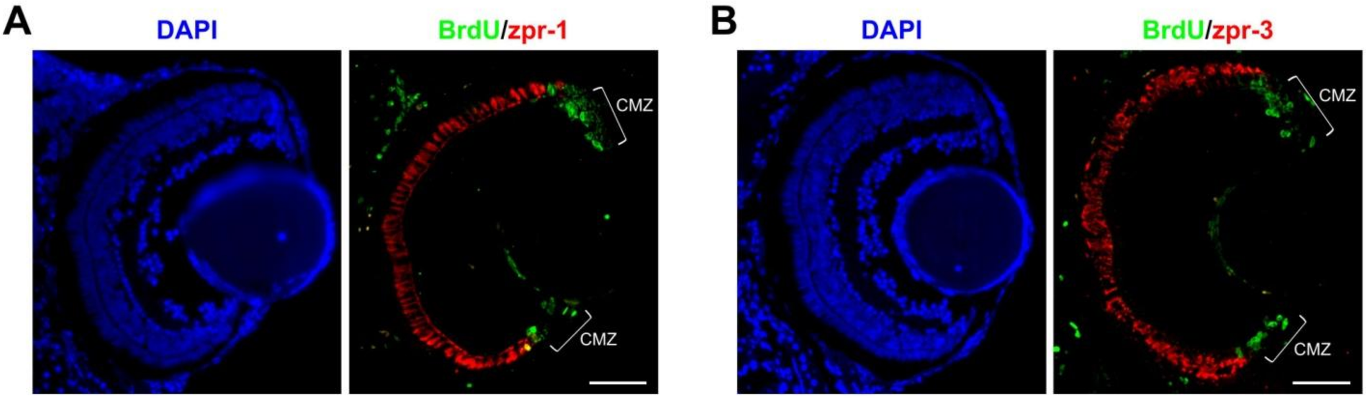

Fig. S3

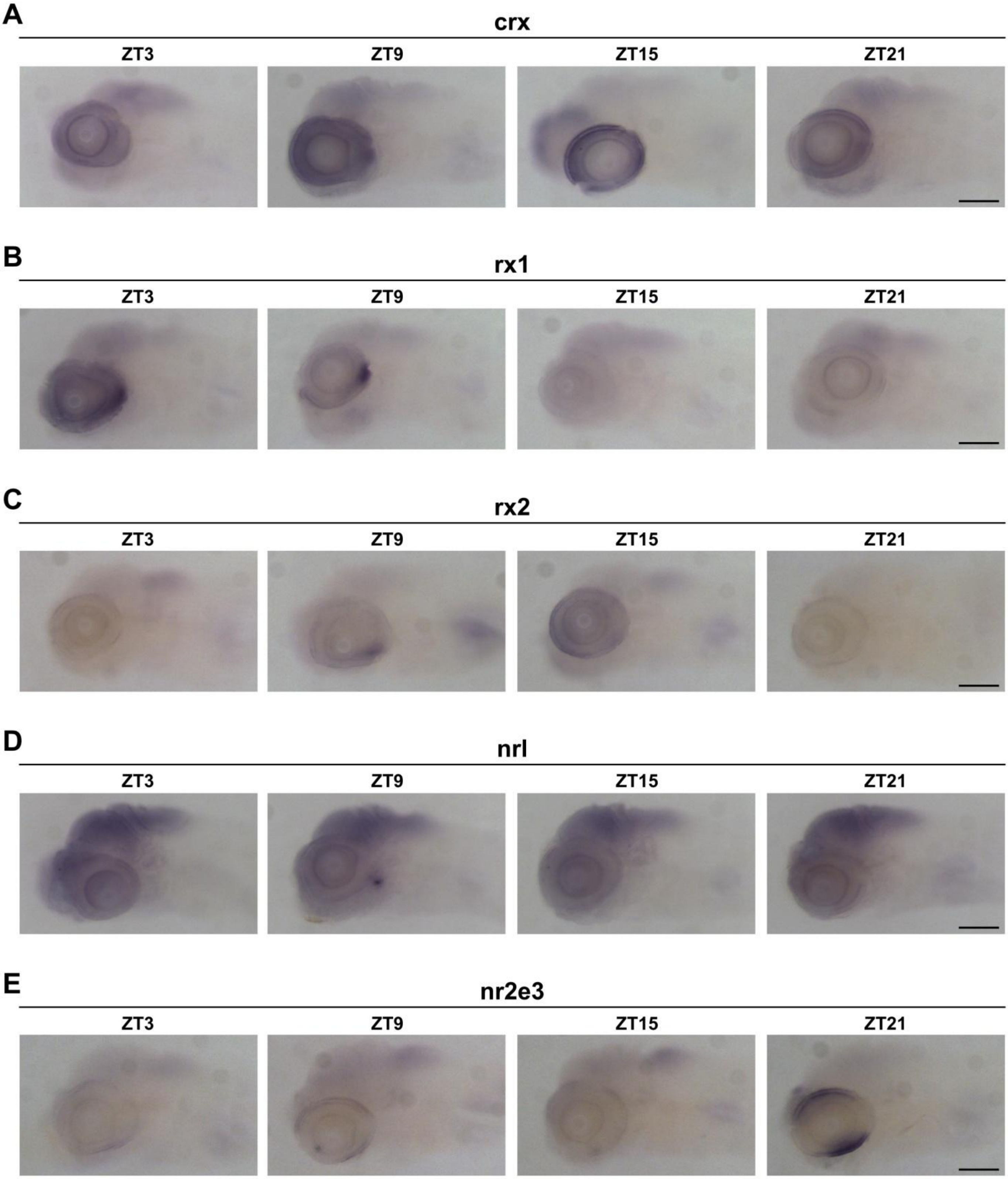
A
crx
ZT3
ZT9
ZT15
ZT21
B
rx1
ZT3
ZT9
ZT15
ZT21
C
rx2
ZT3
ZT9
ZT15
ZT21
D
nrl
ZT3
ZT9
ZT15
ZT21
E
nr2e3
ZT3
ZT9
ZT15
ZT21

**Fig. S4**

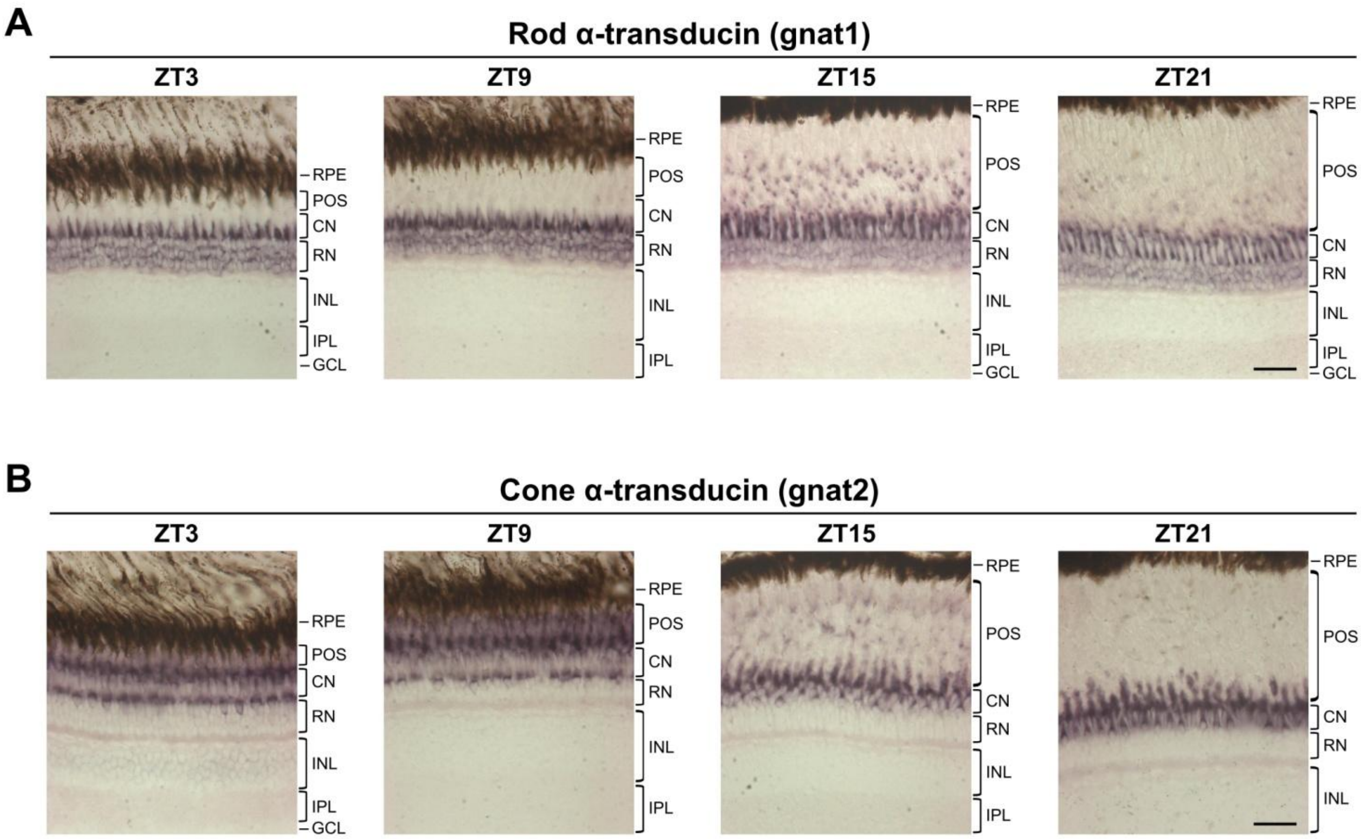
A
Rod α-transducin (gnat1)
ZT3
ZT9
ZT15
ZT21
RPE
POS
CN
RN
INL
IPL
GCL
B
Cone α-transducin (gnat2)
ZT3
ZT9
ZT15
ZT21
RPE
POS
CN
RN
INL
IPL
GCL

**Fig. S5**

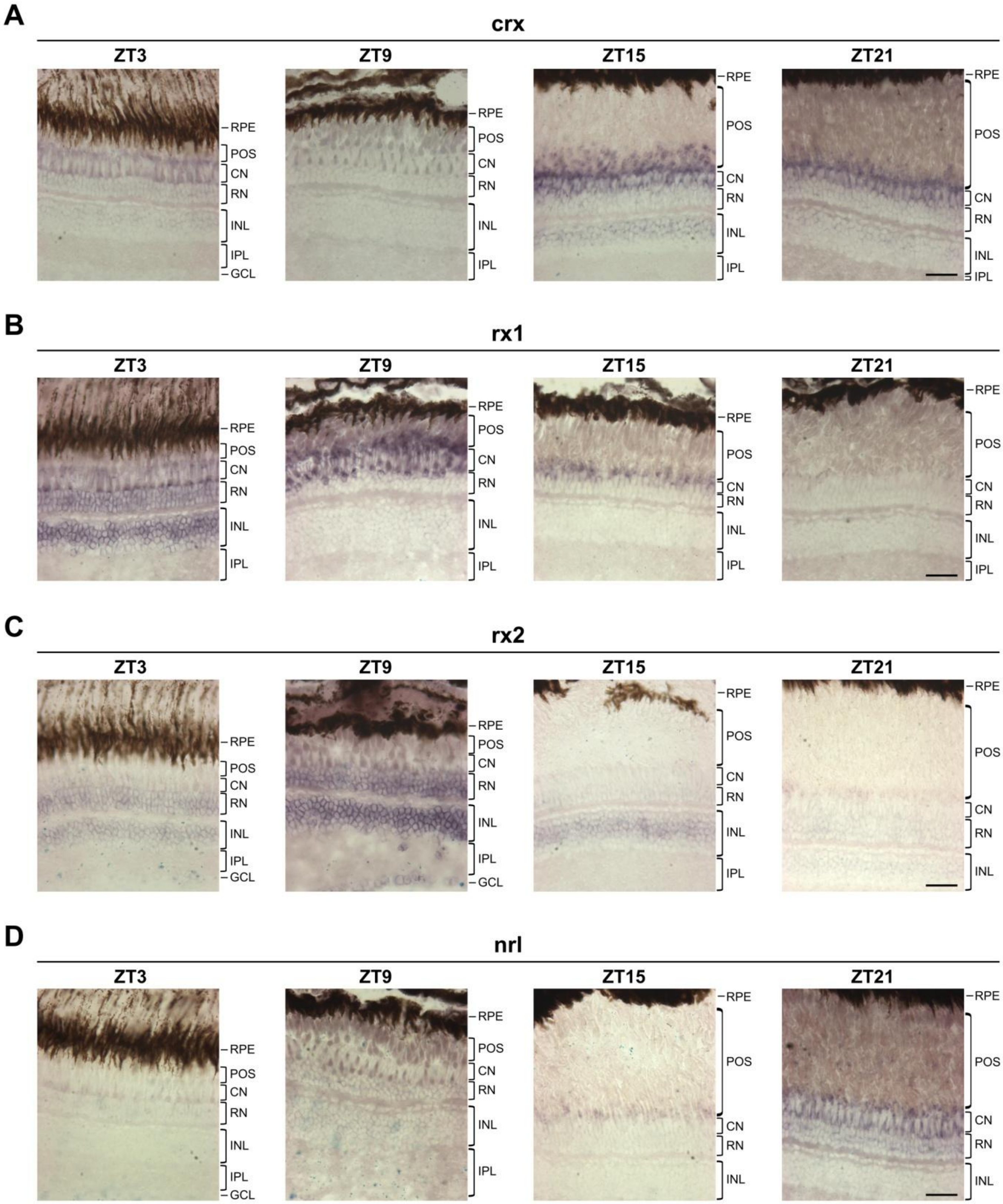

Fig. S6

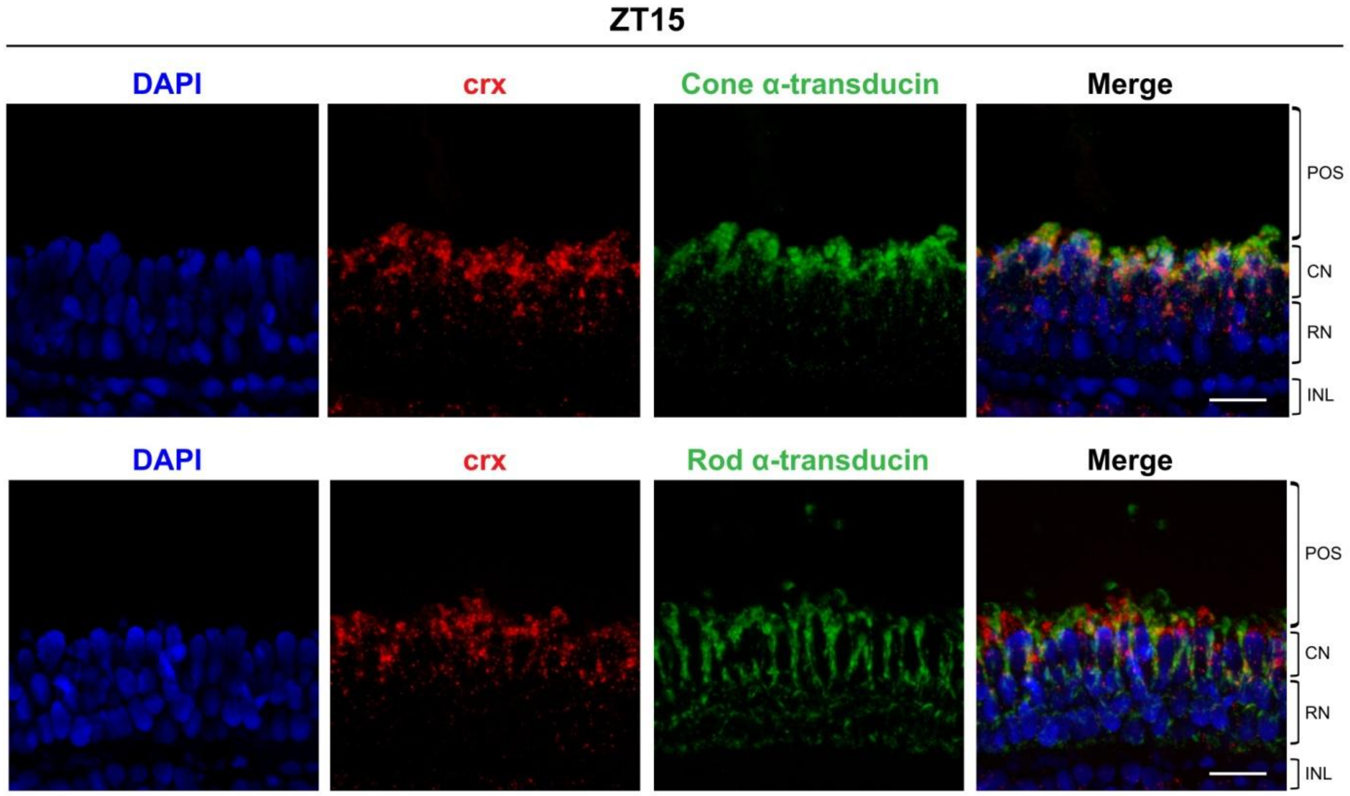
ZT15
DAPI
crx
Cone α-transducin
Merge
POS
CN
RN
INL
DAPI
crx
Rod α-transducin
Merge
POS
CN
RN
INL

**Fig. S7**

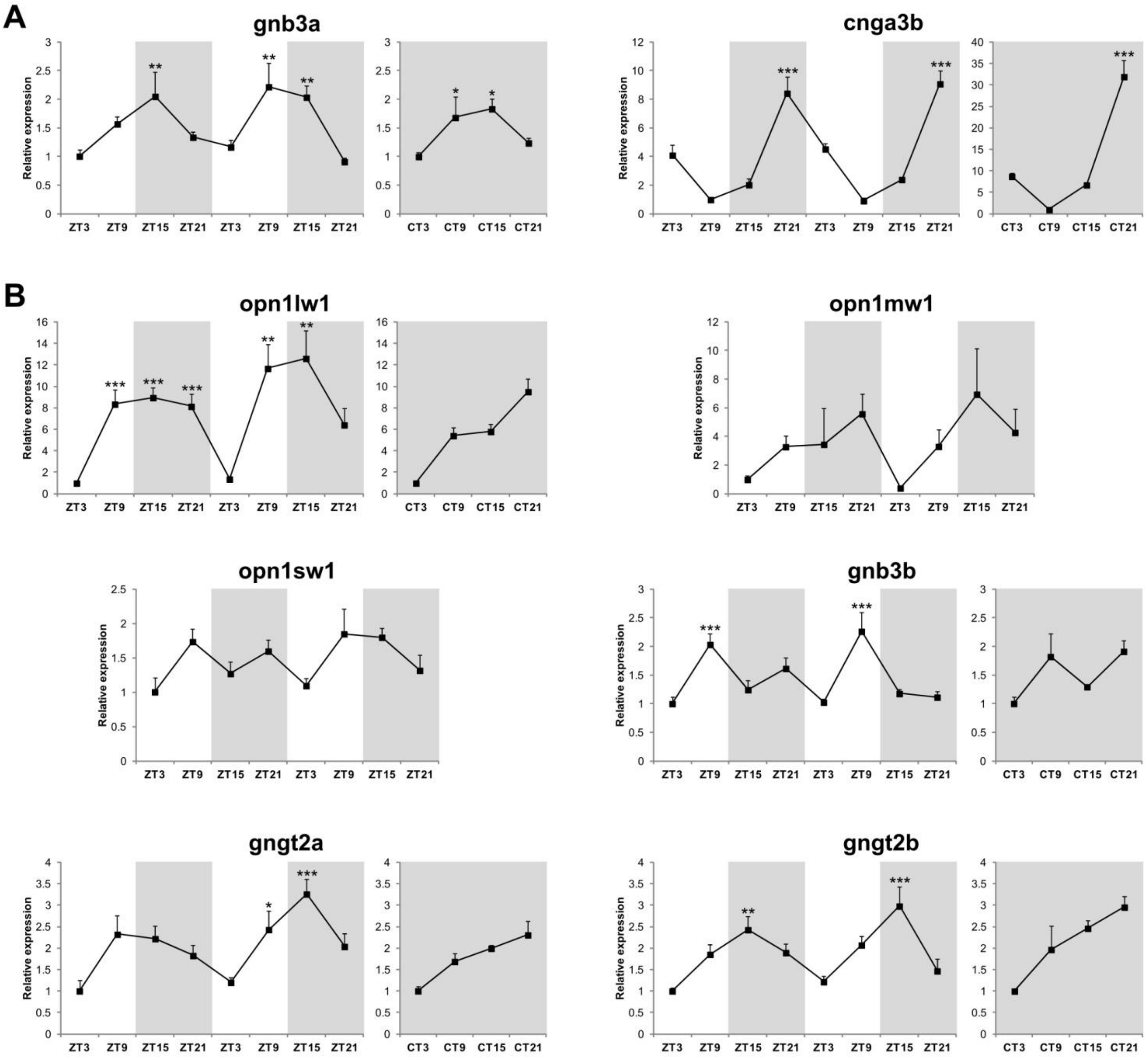
A
gnb3a
cnga3b
B
opn1lw1
opn1mw1
opn1sw1
gnb3b
gngt2a
gngt2b
Relative expression
ZT3 ZT9 ZT15 ZT21 ZT3 ZT9 ZT15 ZT21
CT3 CT9 CT15 CT21

**Fig. S8**

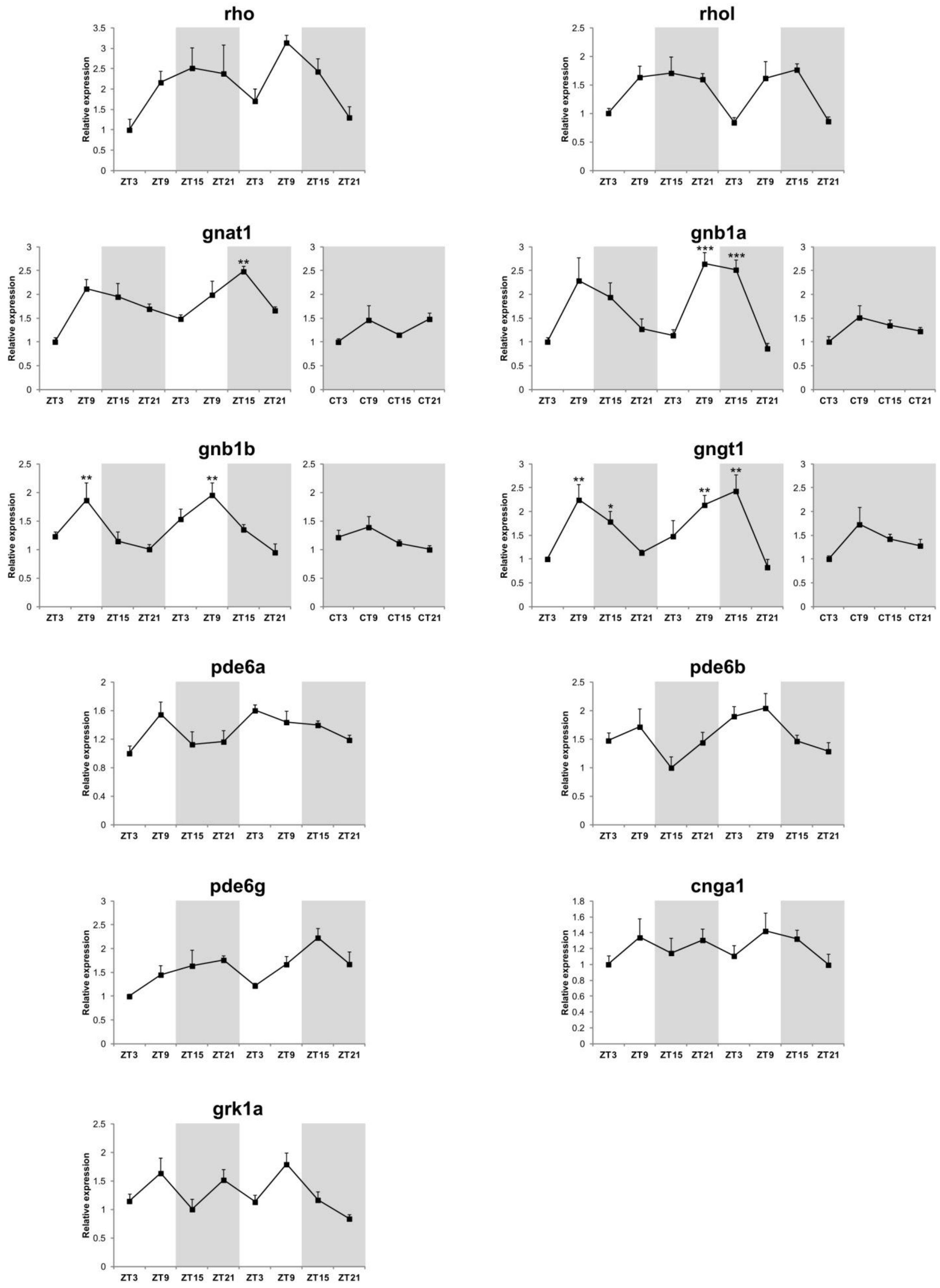
rho
rhol
gnat1
gnb1a
gnb1b
gngt1
pde6a
pde6b
pde6g
cnga1
grk1a
Relative expression
ZT3 ZT9 ZT15 ZT21 ZT3 ZT9 ZT15 ZT21
CT3 CT9 CT15 CT21
*
**
***

**Table S1. Primers used for qPCR analysis.**

| Gene | Forward primer (5'-3') | Reverse primer (5'-3') | Product size (bp) |
|---|---|---|---|
| ***arr3a*** | GAGGAGAAGATCGCCCATCG | CTGGCCATTTCCACTGGTCT | 93 |
| ***arr3b*** | GCATGTGCCTTTCGCTATGG | CTGCATTGGTGTGTTTGGGG | 129 |
| ***cnga1*** | ATCGCAGAACCGCCAACATA | CGGATATTCAGTCAGCGCCT | 92 |
| ***cnga3a*** | ATGAGACACGAATCAGCCCA | TCCTGCTCAGCGTGATGTAA | 119 |
| ***cnga3b*** | ATCAGTGACCCCGAGTTTGG | AATGGTGGTGAGCGTGAGAG | 81 |
| ***cngb3*** | CCAACCTCTTCGTGCTGGAT | TGCCTTTGGCCTTCATCAGT | 111 |
| ***crx*** | ATCCACTGTGTGGTTCAGGC | GCTGTAGGAAGAGGGCTGAC | 83 |
| ***eef1a1l1*** | CAGCTGATCGTTGGAGTCAA | TGTATGCGCTGACTTCCTTG | 94 |
| ***gnat1*** | CCGTTACTTCGCCACCACAT | GAAGGTGTTGGGACCGTCAT | 121 |
| ***gnat2*** | CGTGATCTGAGGTACAGGGC | GCTACCCATCTCGTCGTCTG | 122 |
| ***gnb1a*** | TGCCACCCTCTCTCAGATCA | CAAGTGTCCCCTCAGTGTCC | 85 |
| ***gnb1b*** | AGATGCCAATCATGCGTTTTGT | CTTTCACAGGAGGGCGCATA | 105 |
| ***gnb3a*** | GGTGAAATGGAGCAACTGCG | GTGTAAGGTCCTGCACGGTT | 85 |
| ***gnb3b*** | GCTGTTCCCCTGAAGTCCTC | ACATGTTATCCAGTCCGCCG | 88 |
| ***gngt1*** | TCCAGAGGAGAAGAACCCGT | ACTGCGAATGTTGGGGGATT | 83 |
| ***gngt2a*** | TCAGTTCATCGCCAGACCAC | GGCCATCTTGCCCTATTGGT | 105 |
| ***gngt2b*** | GCCAGAGGACAAGAACCCAT | AAGCATTTCTAGGACCGGCA | 129 |
| ***grk1a*** | GCTGTACGCCTGCAAGAAAC | TCCACCATCGCTCCCTCATA | 72 |
| ***grk7a*** | AGGACGAGTCTGGACAAGGA | GTACTGGCGGAACAGCTTCT | 75 |
| ***neuroD*** | CGGTCTTCCCAGCCCTCCGT | GAACGGCTCCAGTGCGCTCC | 91 |
| ***nr2e3*** | TGGAAAGGTCCTGAACACGG | CTGCGCTTGAAAAAGCCACT | 111 |
| ***nrl*** | GCTTCCAGCGATGTCTTCCT | GGGGTGTCAGGCTTTACCTC | 114 |
| ***opn1lw1*** | AGATGCAATTTATGCAGCCCG | CATCGAGGGGCAATGTGGTA | 126 |
| ***opn1mw1*** | ACACCCTTTTCTGTGGCAAG | ATGACGGAGCACTGAATAGGC | 120 |
| ***opn1sw1*** | TTCCAAAGTCAGCCCCTTCG | GTTCATAGGTGTGCCCACGA | 112 |
| ***pde6a*** | CAGTCAACAAGATCGGGGCT | GCTCAGGTGAAACACTCGGA | 104 |
| ***pde6b*** | TGGCCTCCAAATTCCCGAAA | AGCATTGTGGCTTGCACTTG | 80 |
| ***pde6c*** | CACAGTTCCTGGGATGGTCC | CGGAGTGGCTTTGGTCTGAT | 116 |
| ***pde6g*** | TTCAAGAGCAAGCCCCCAAA | AGCTCCAGATGGTTGAAGGC | 122 |
| ***pde6h*** | AGAGAGGAGGACCACCAAAGT | CCATTCCTGGGATGTCGTCT | 105 |
| ***rho*** | ACTTCCGTTTCGGGGAGAAC | GAAGGACTCGTTGTTGACAC | 176 |
| ***rhol*** | GCGGTGGCTGACTTGTTTAT | CTCAACAGCCAGAACAACCA | 165 |
| ***rx1*** | GGACCAGGATTCGTTGCTCA | ATCCCTAAGGGGTGGCAGAT | 130 |
| ***rx2*** | TCCAGCCCACCTATACTGCT | ACTGGTTGGCATTGGTAGGG | 103 |
| ***saga*** | TGTCACATTGTCCTGCGTGT | CTGCCGGGTGGAAATGTAGA | 97 |
| ***sagb*** | TTGTGCTGATCGACCCAGAG | ATATCCCTGCGAAACGCCAT | 118 |
